# Supplementary material for: Novel Chrysin-De-Allyl PAC-1 Hybrid Analogues as Anticancer Compounds: Design, Synthesis, and Biological Evaluation
Source: Molecules. 2020 Jul 4;25(13):3063. doi: 10.3390/molecules25133063 (PMC7412250; doi:10.3390/molecules25133063)
Supplement: Supplementary file 1 [file molecules-25-03063-s001.pdf]

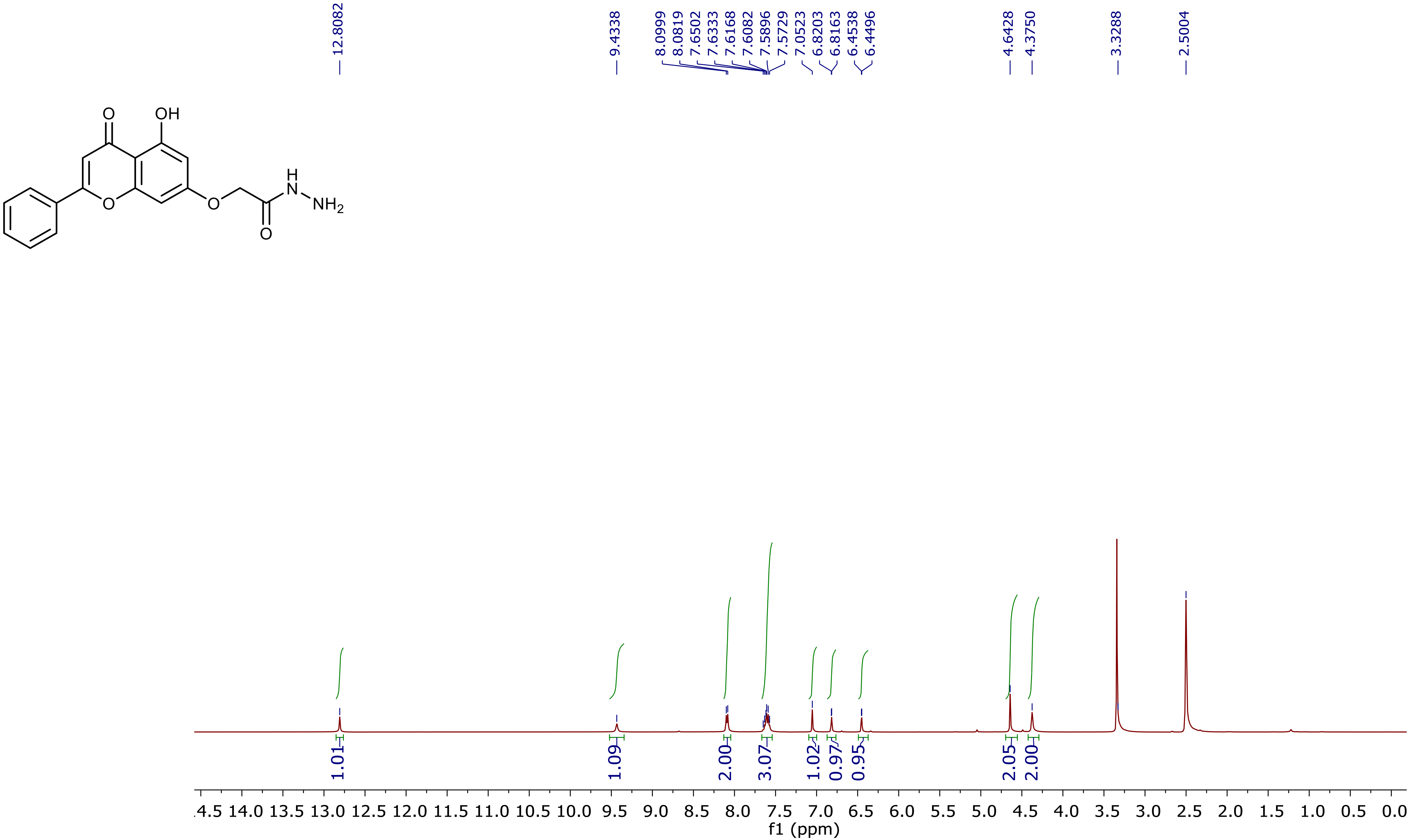

S1. ^{1}H NMR of Compound **3**

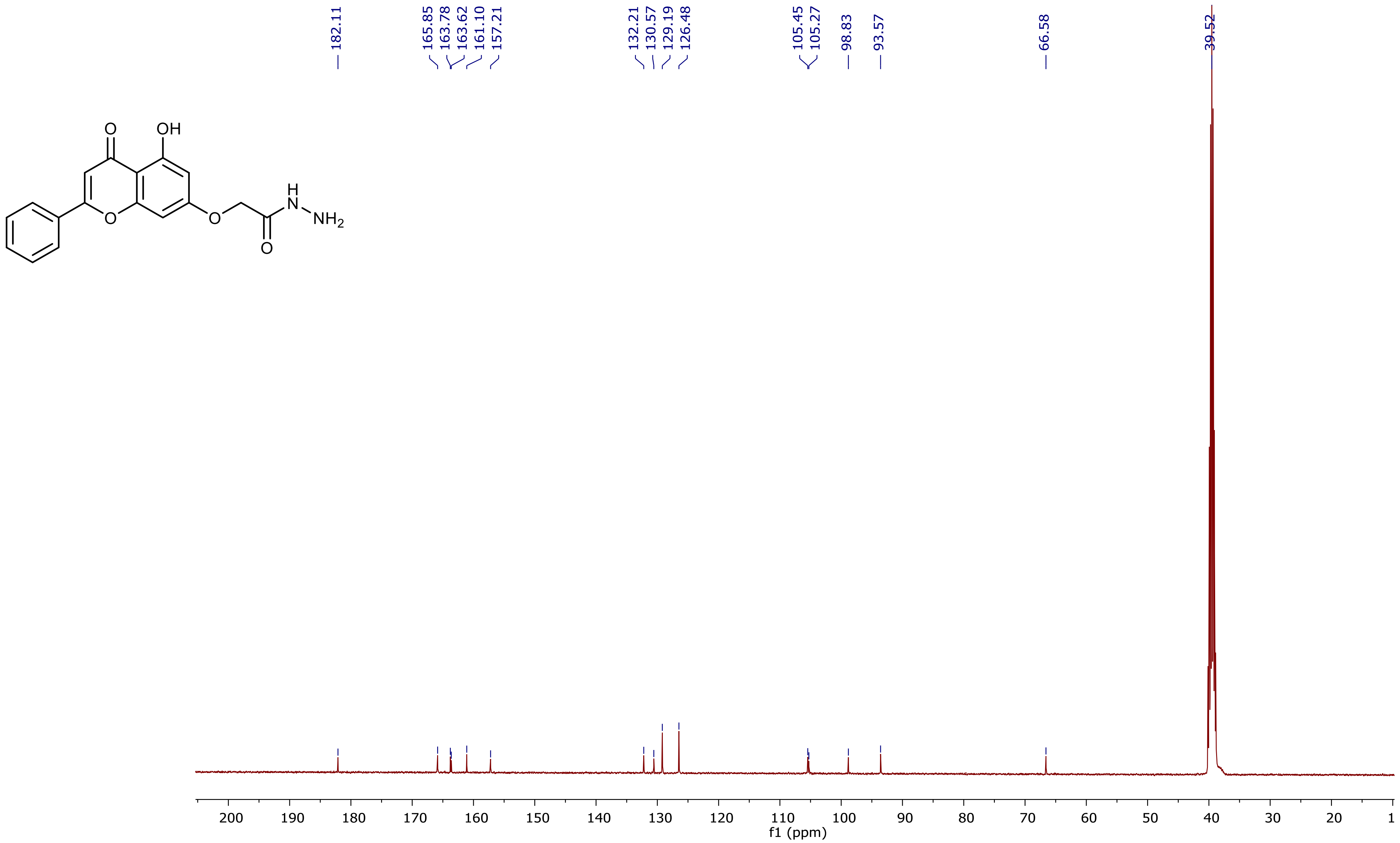

S2. ^{13}C NMR of Compound **3**

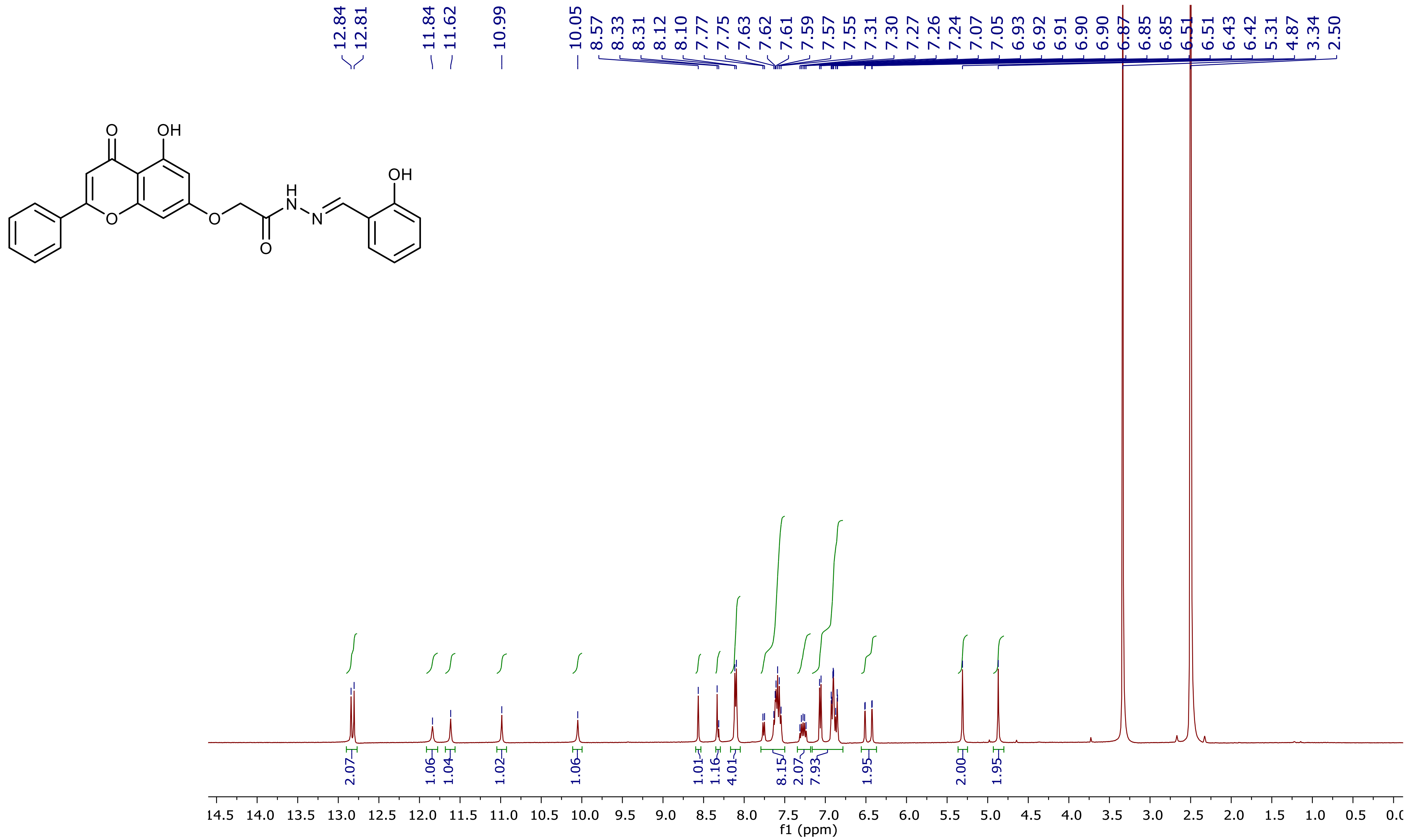

S3. ^{1}H NMR of Compound **4a**

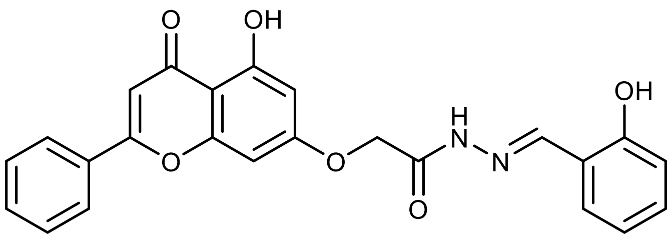

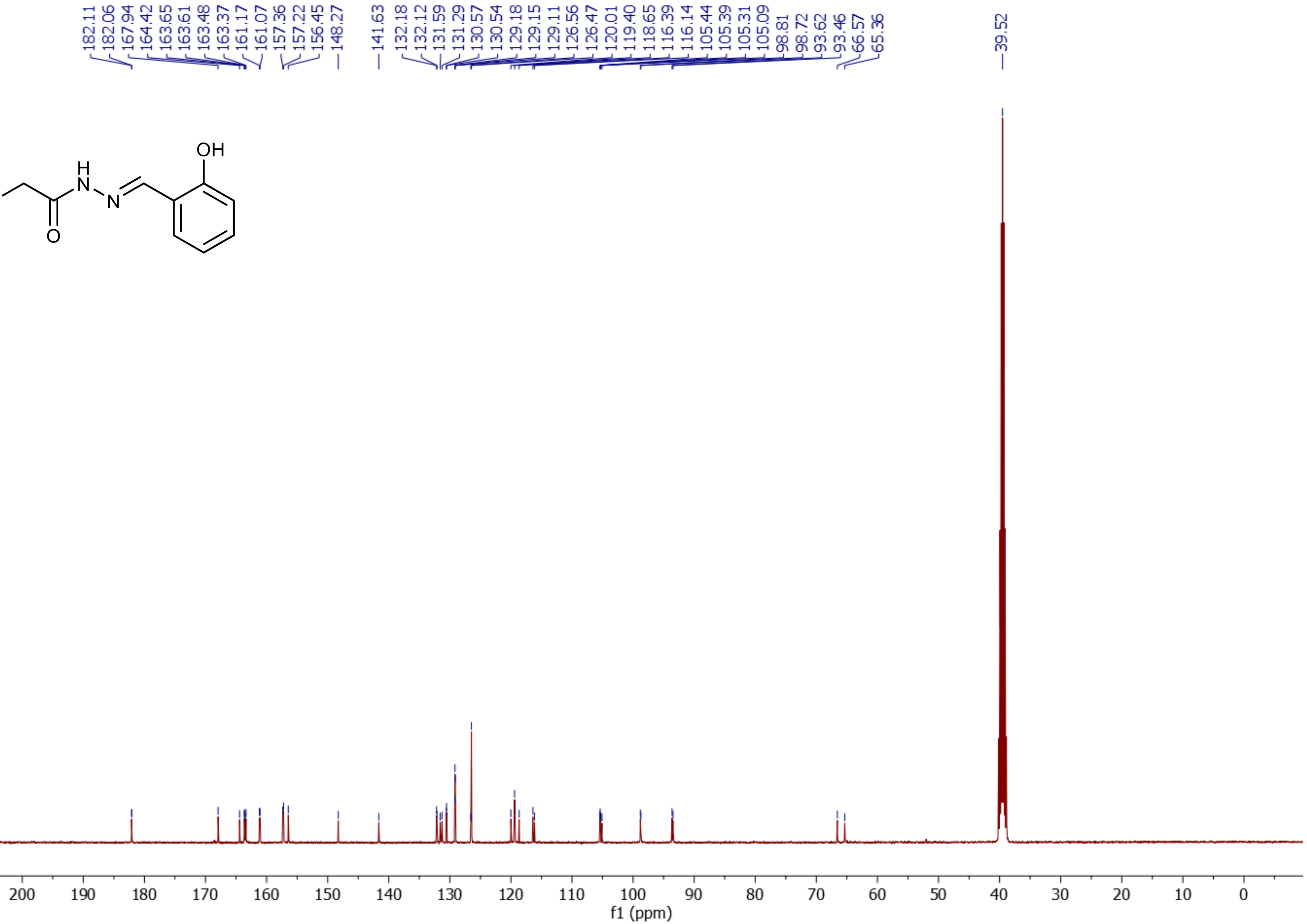

S4. ^{13}C NMR of Compound **4a**

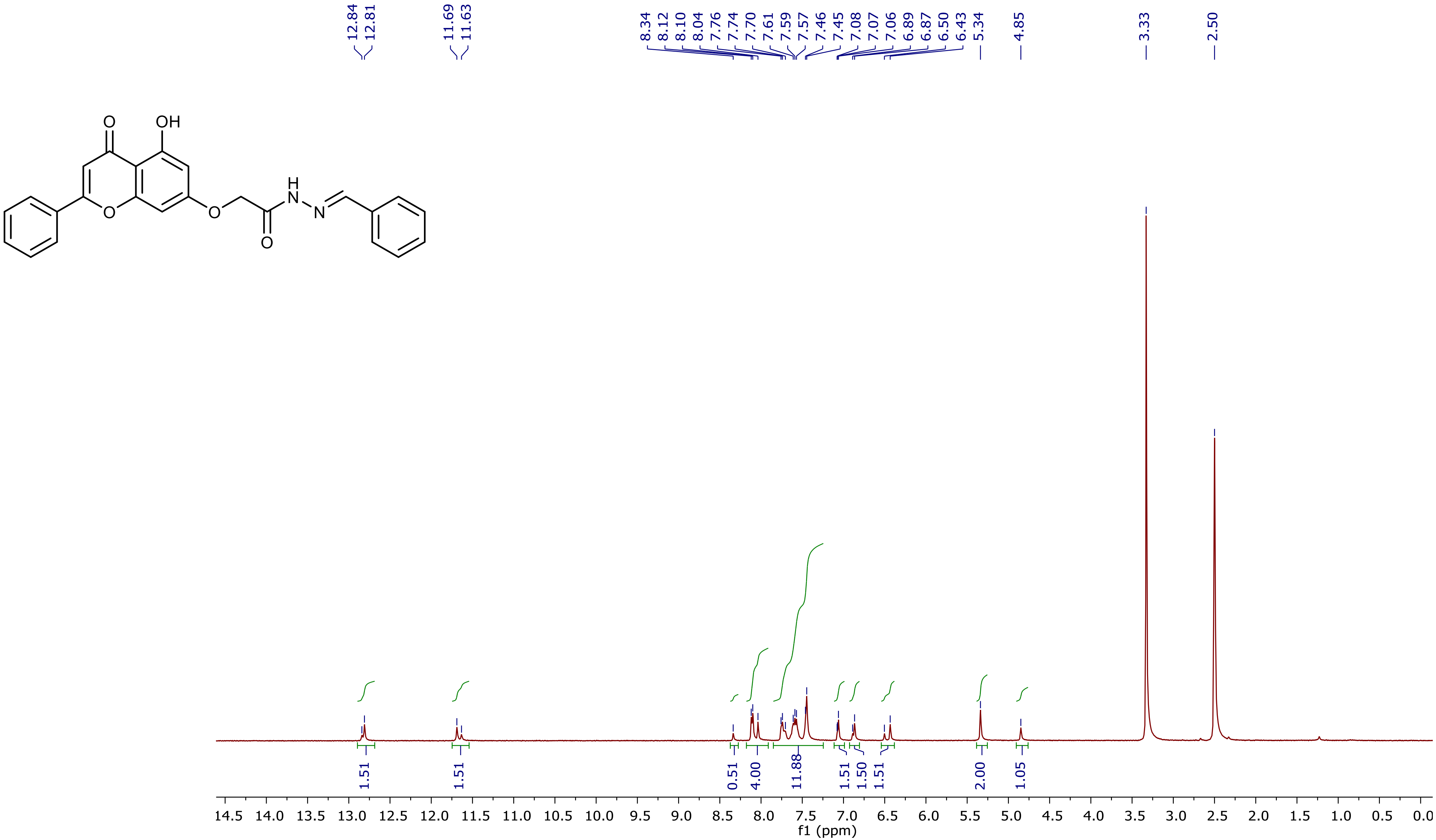

S5. ^{1}H NMR of Compound **4b**

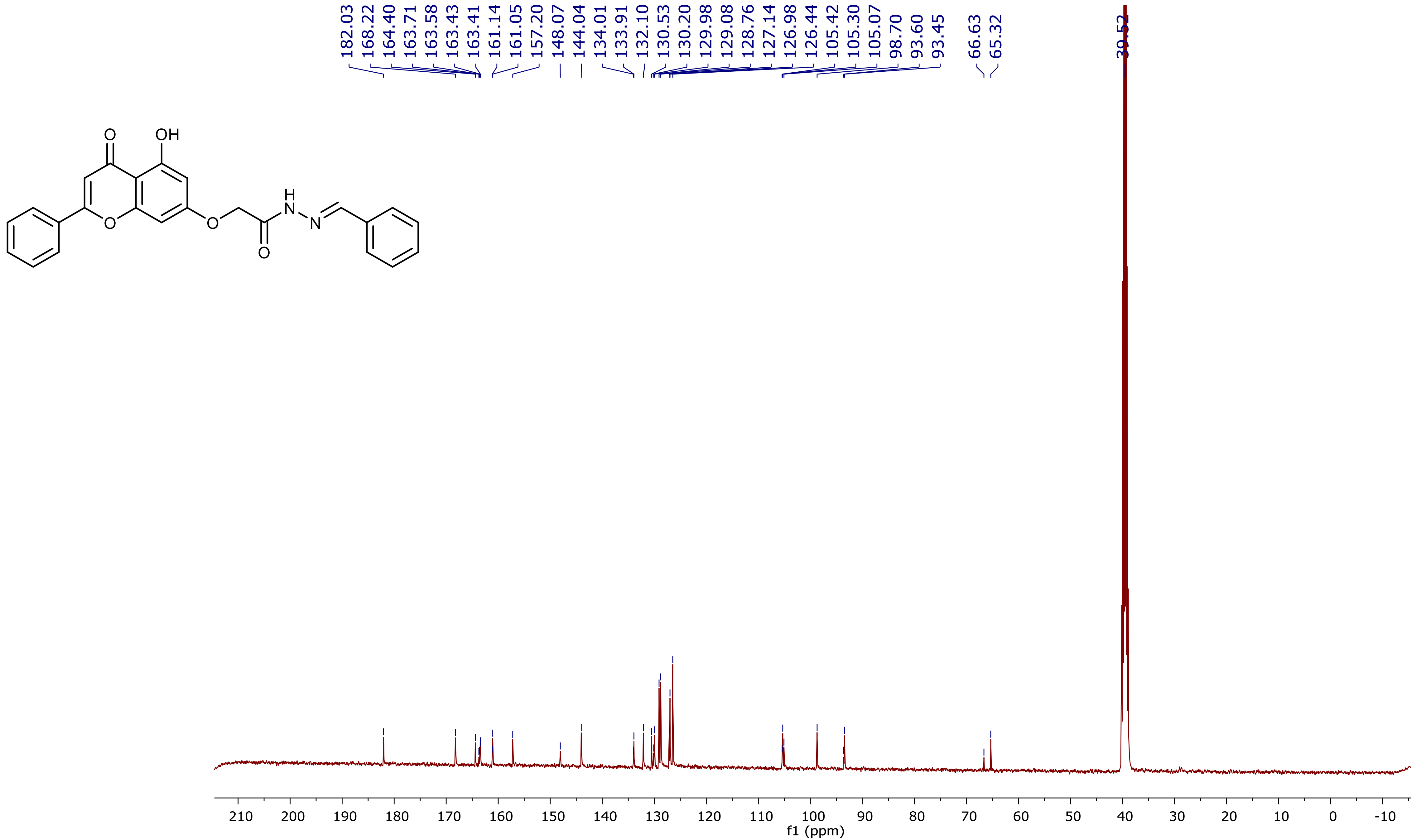

S6. ^{13}C NMR of Compound **4b**

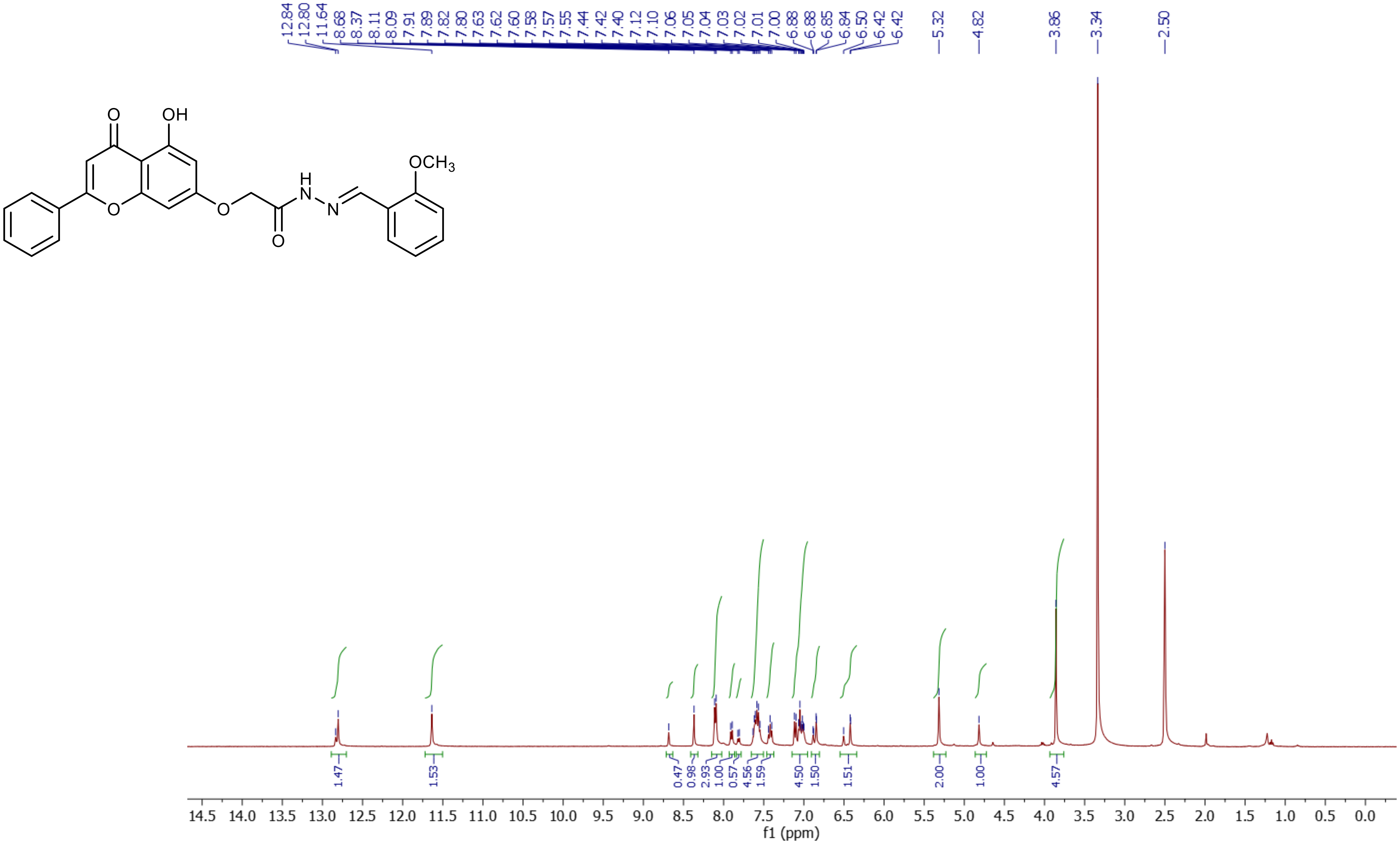

S7. ^{1}H NMR of Compound **4c**

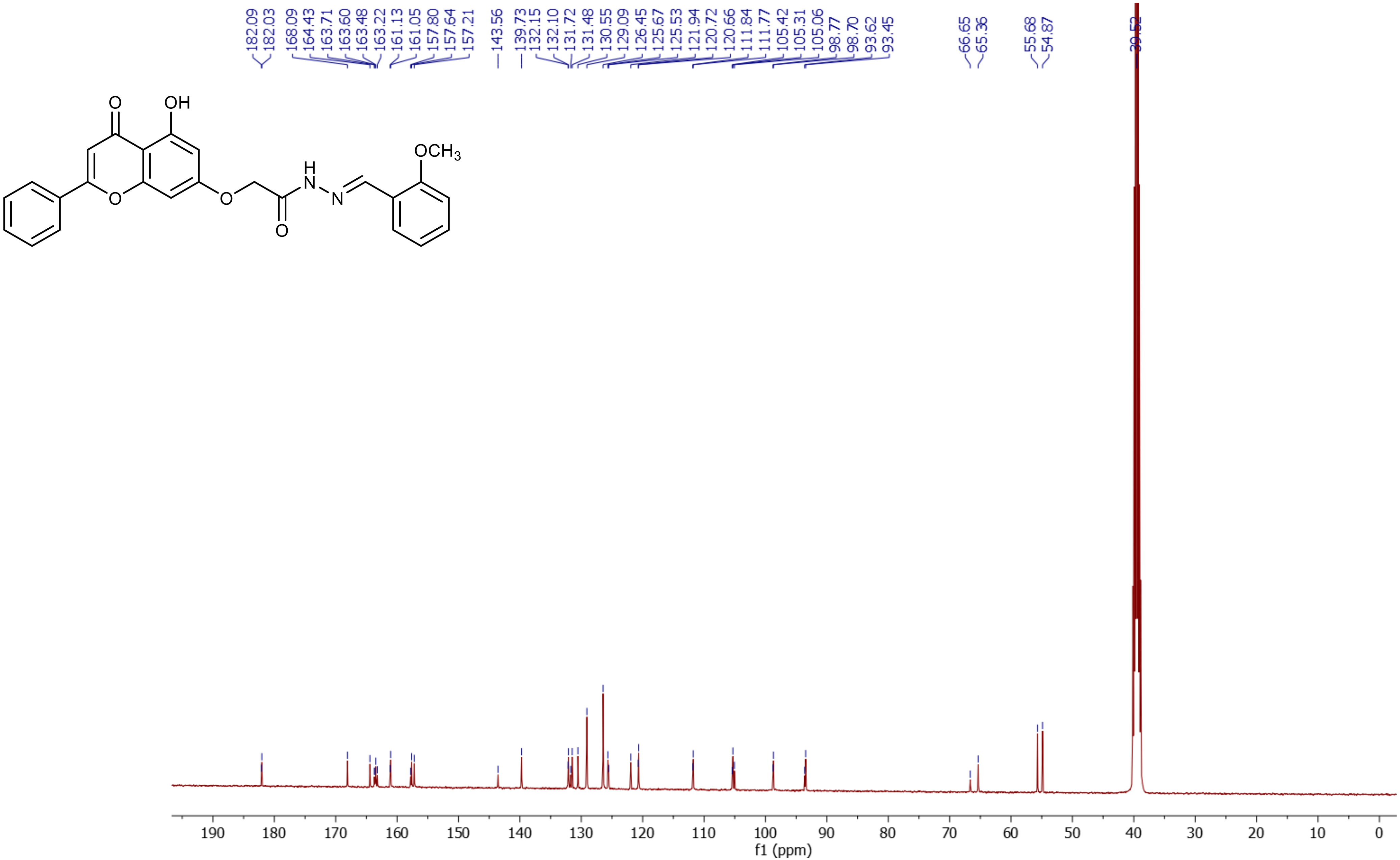

S8. ^{13}C NMR of Compound **4c**

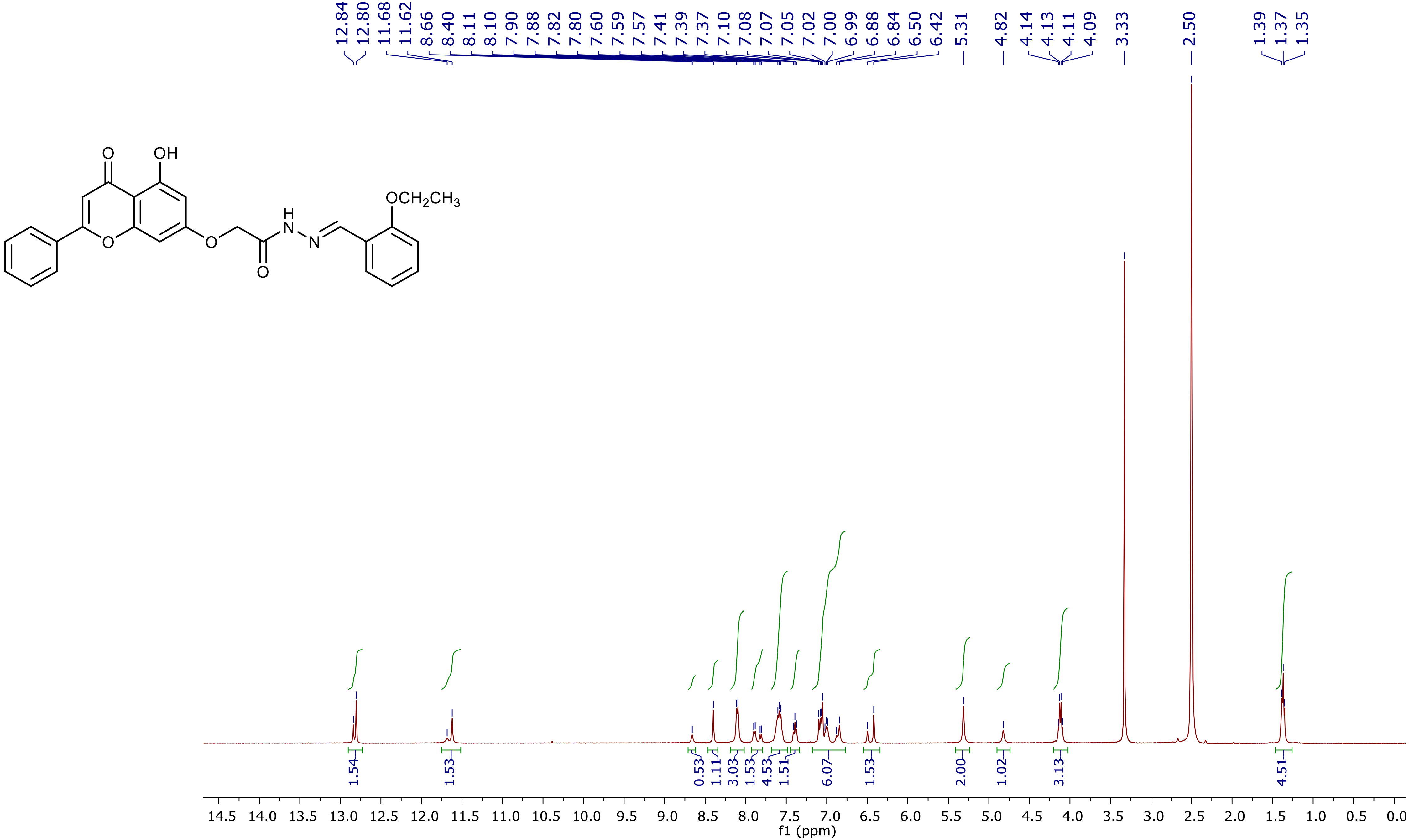

S9. ^{1}H NMR of Compound **4d**

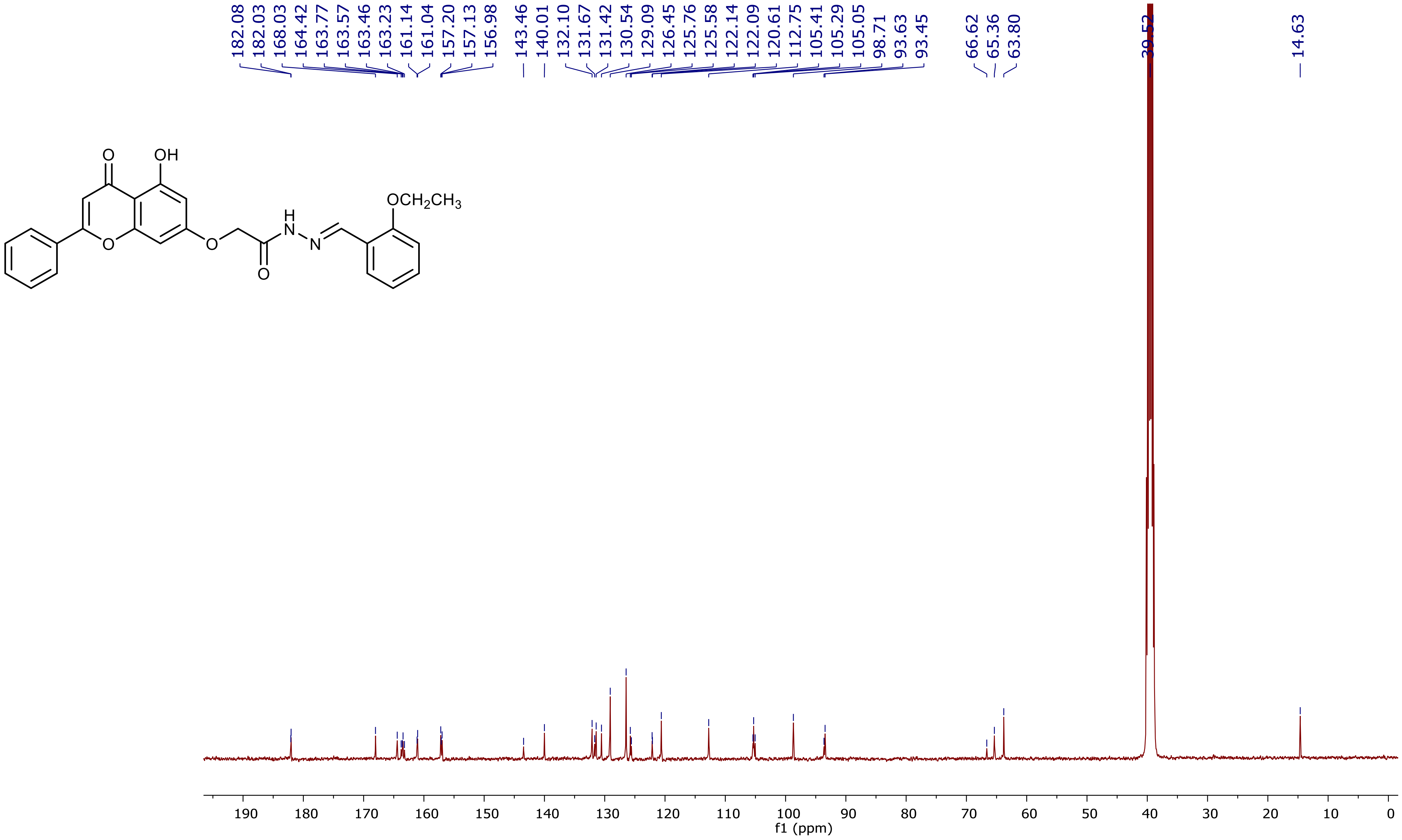

S10. ^{13}C NMR of Compound **4d**

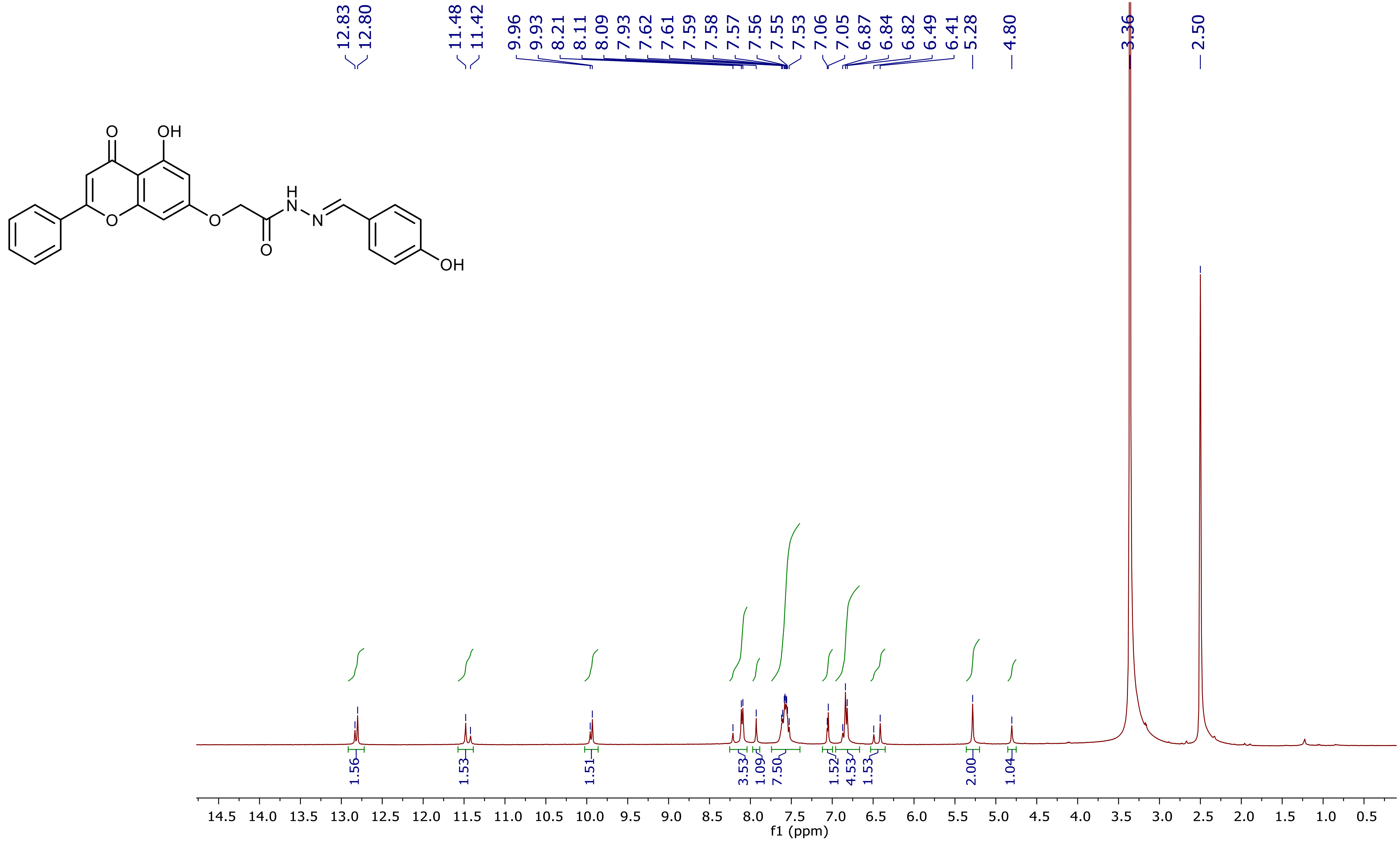

S11. ^{1}H NMR of Compound **4e**

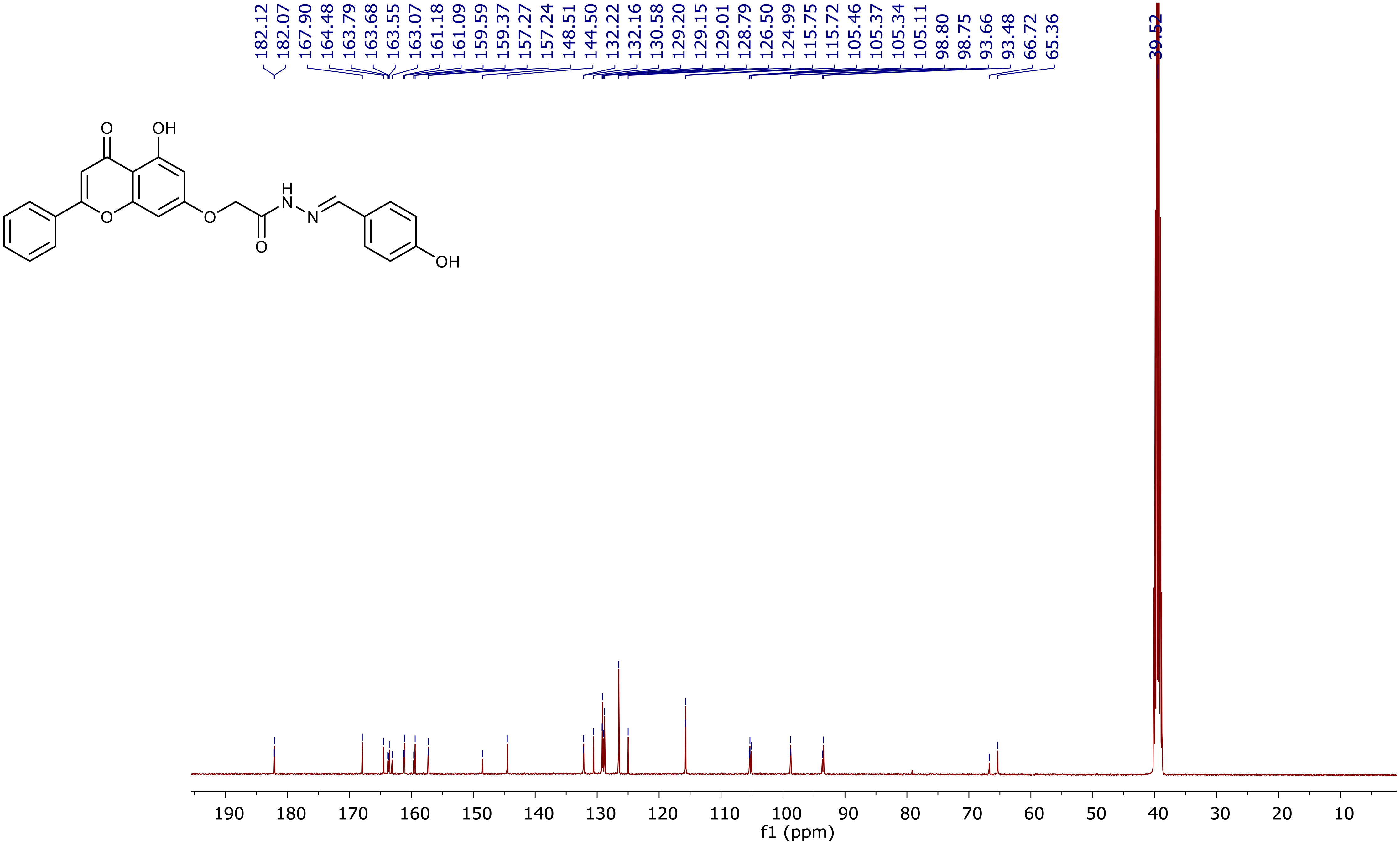

S12. ^{13}C NMR of Compound **4e**

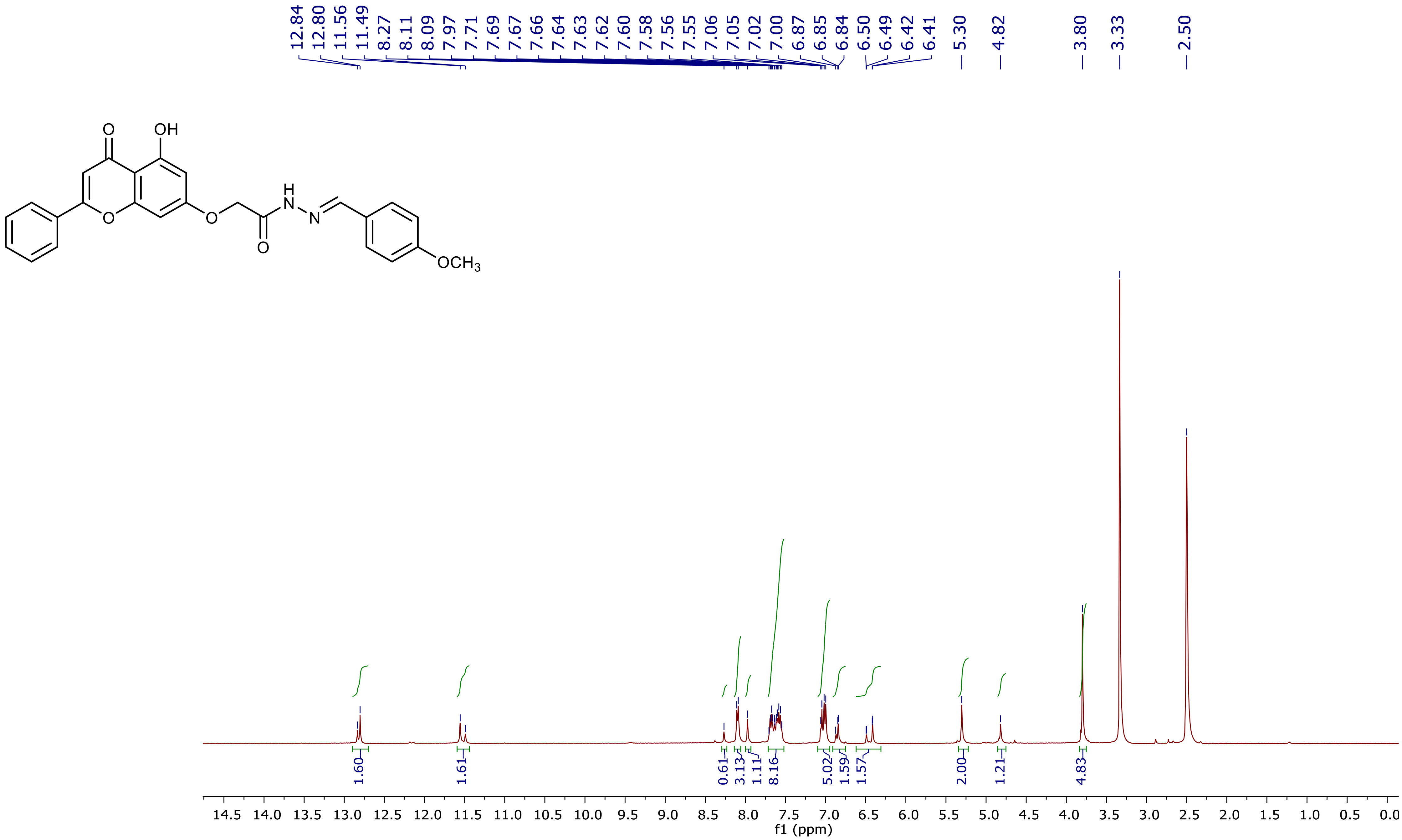

S13. ^{1}H NMR of Compound **4f**

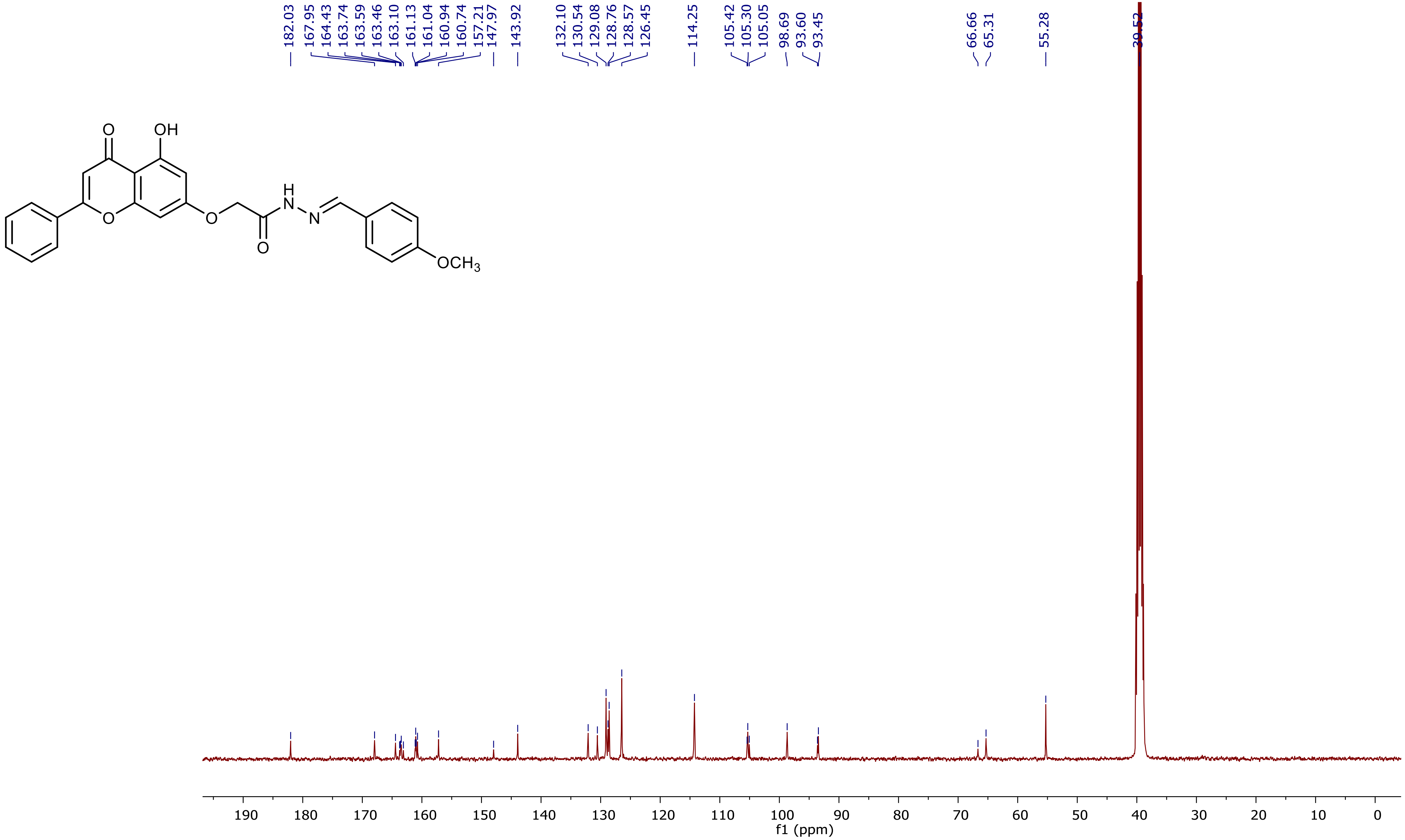

S14. ^{13}C NMR of Compound **4f**

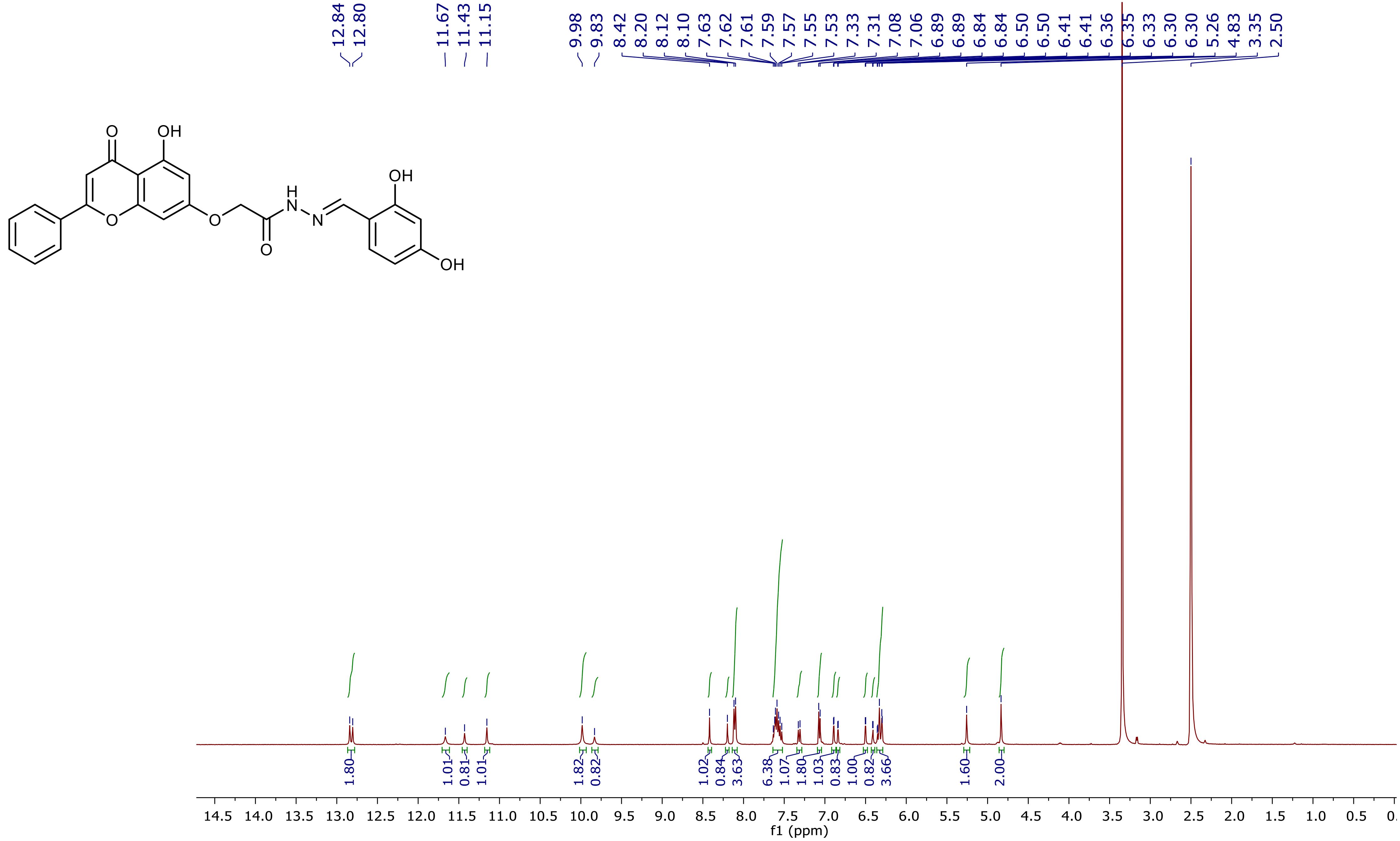

S15. ^{1}H NMR of Compound **4g**

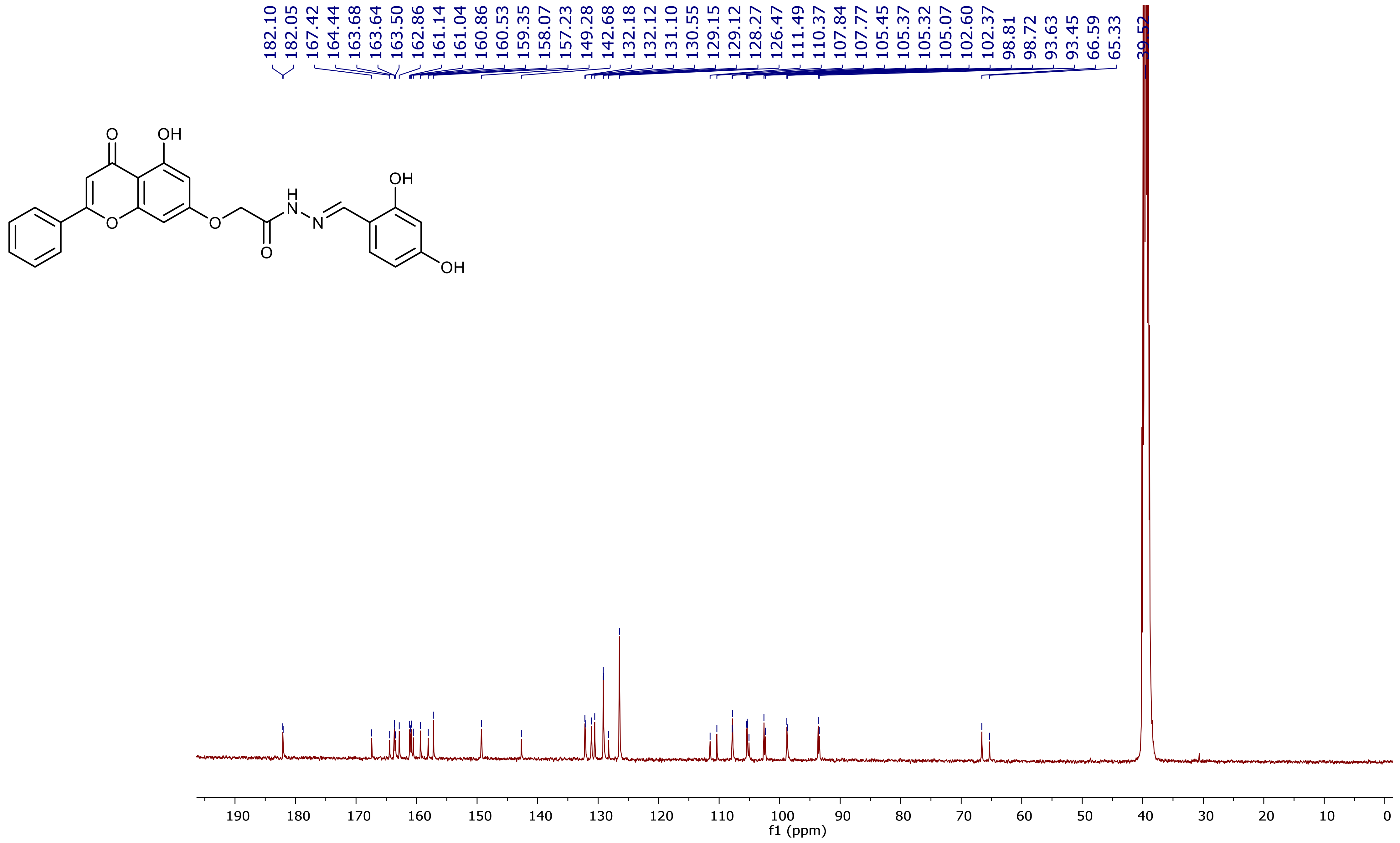

S16. ^{13}C NMR of Compound **4g**

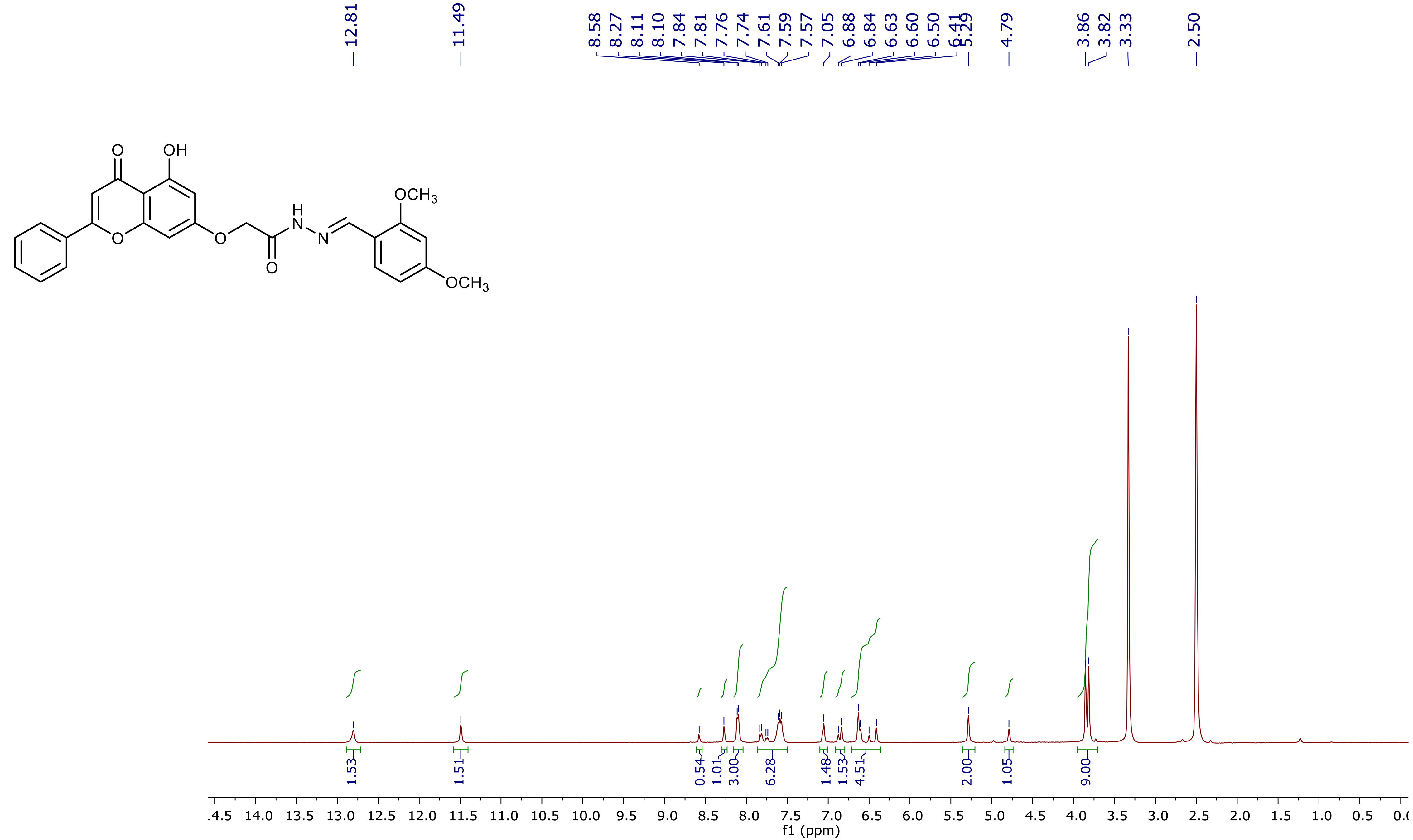

S17. ^{1}H NMR of Compound **4h**

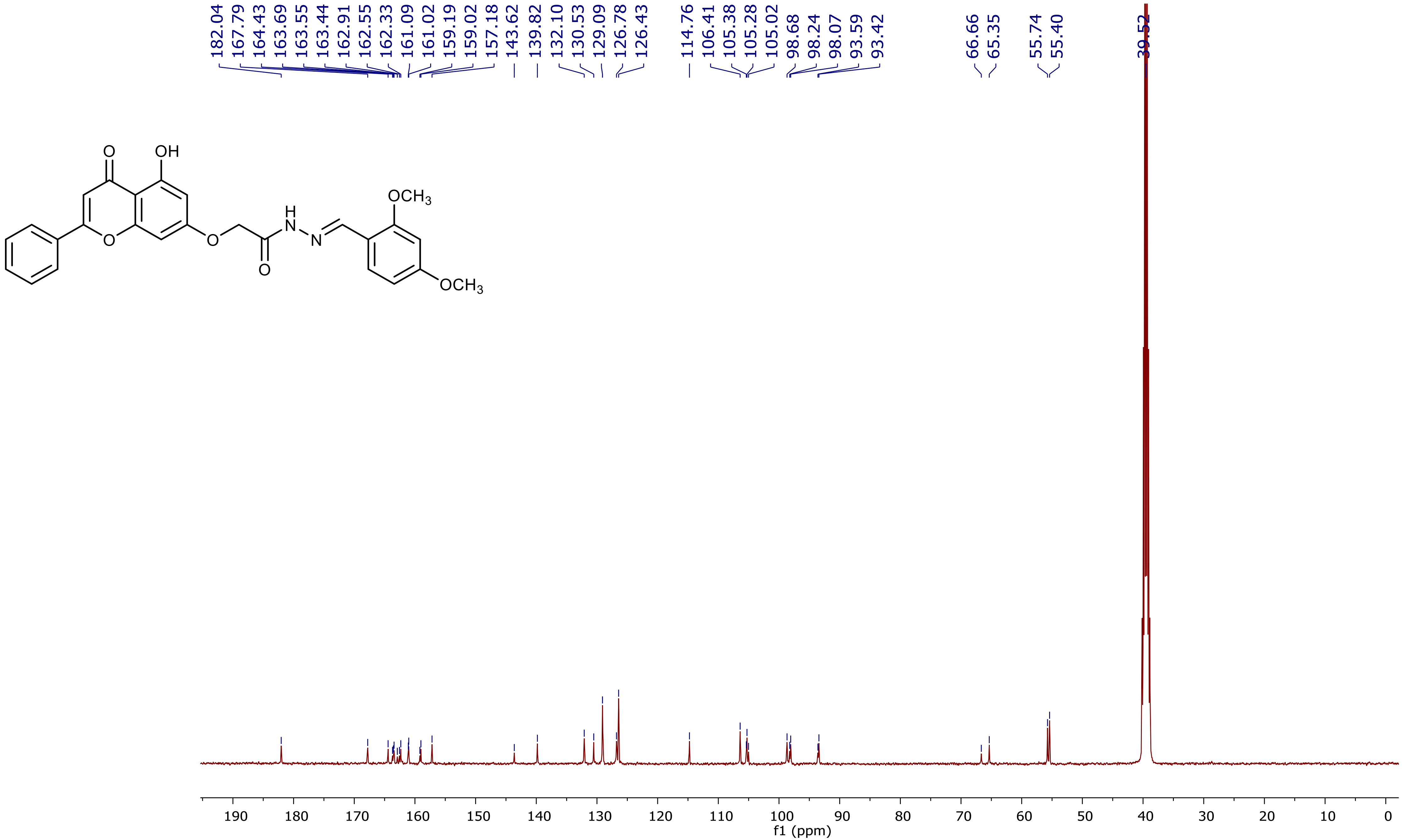

S18. ^{13}C NMR of Compound **4h**

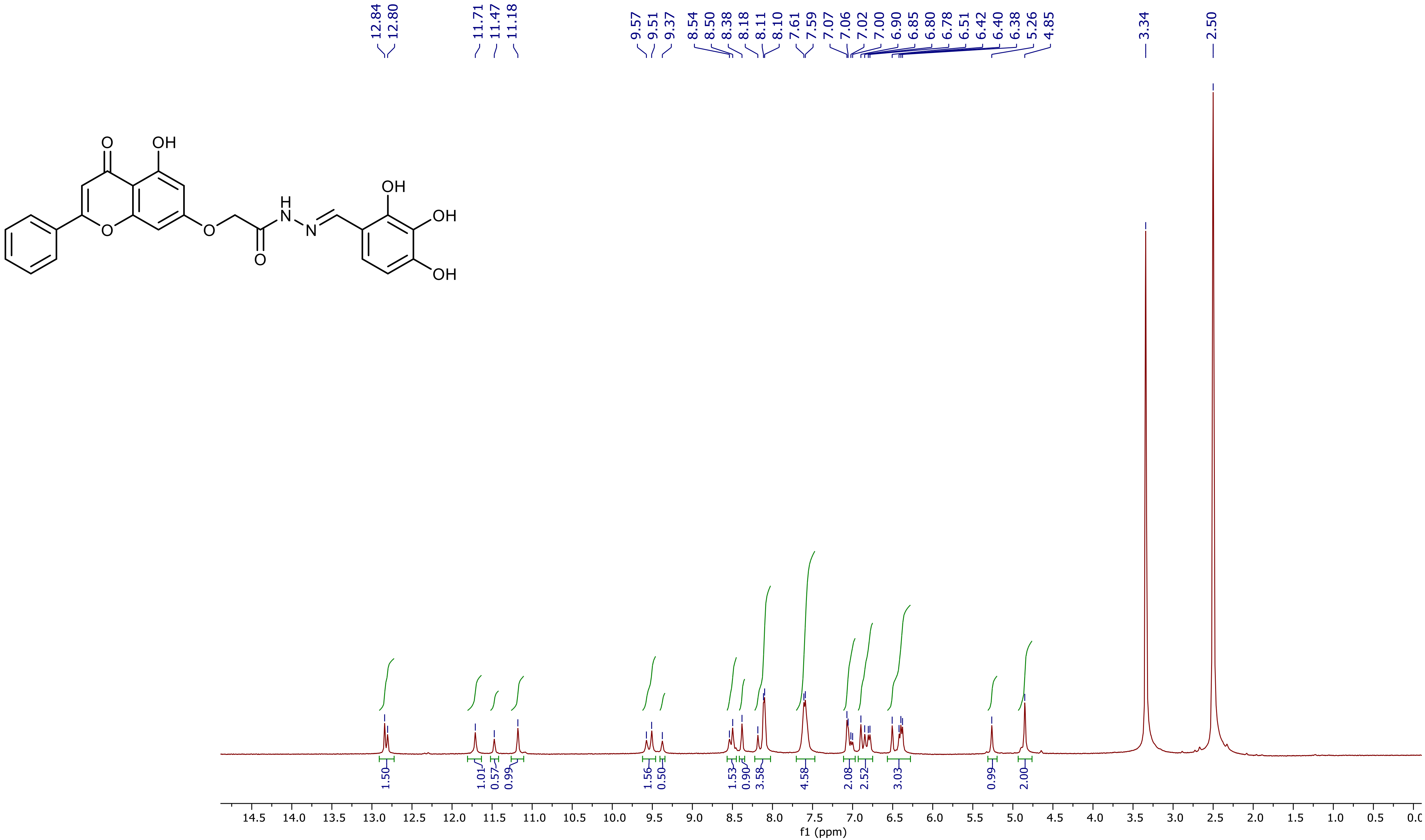

S19. ^{1}H NMR of Compound **4i**

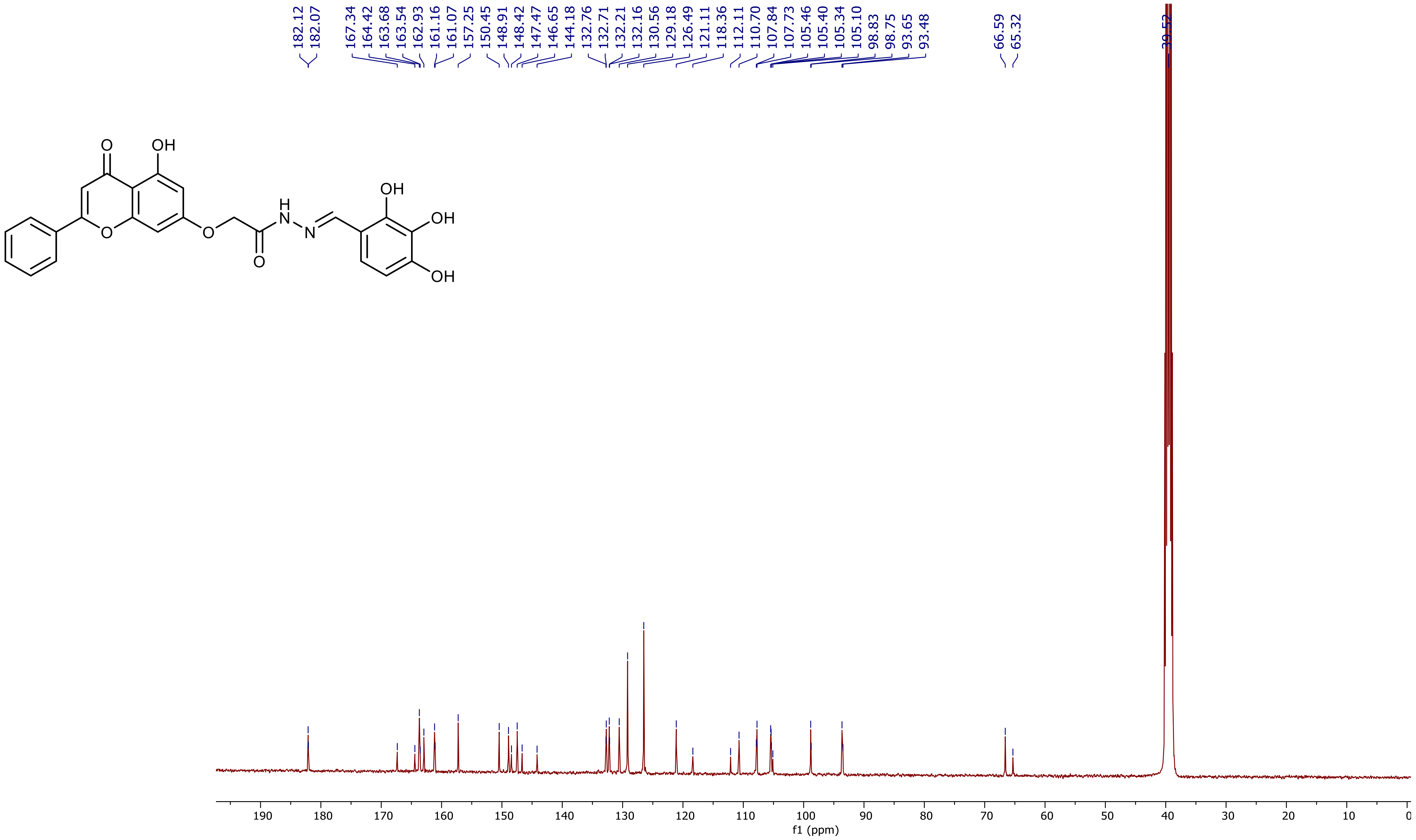

S20. ^{13}C NMR of Compound **4i**

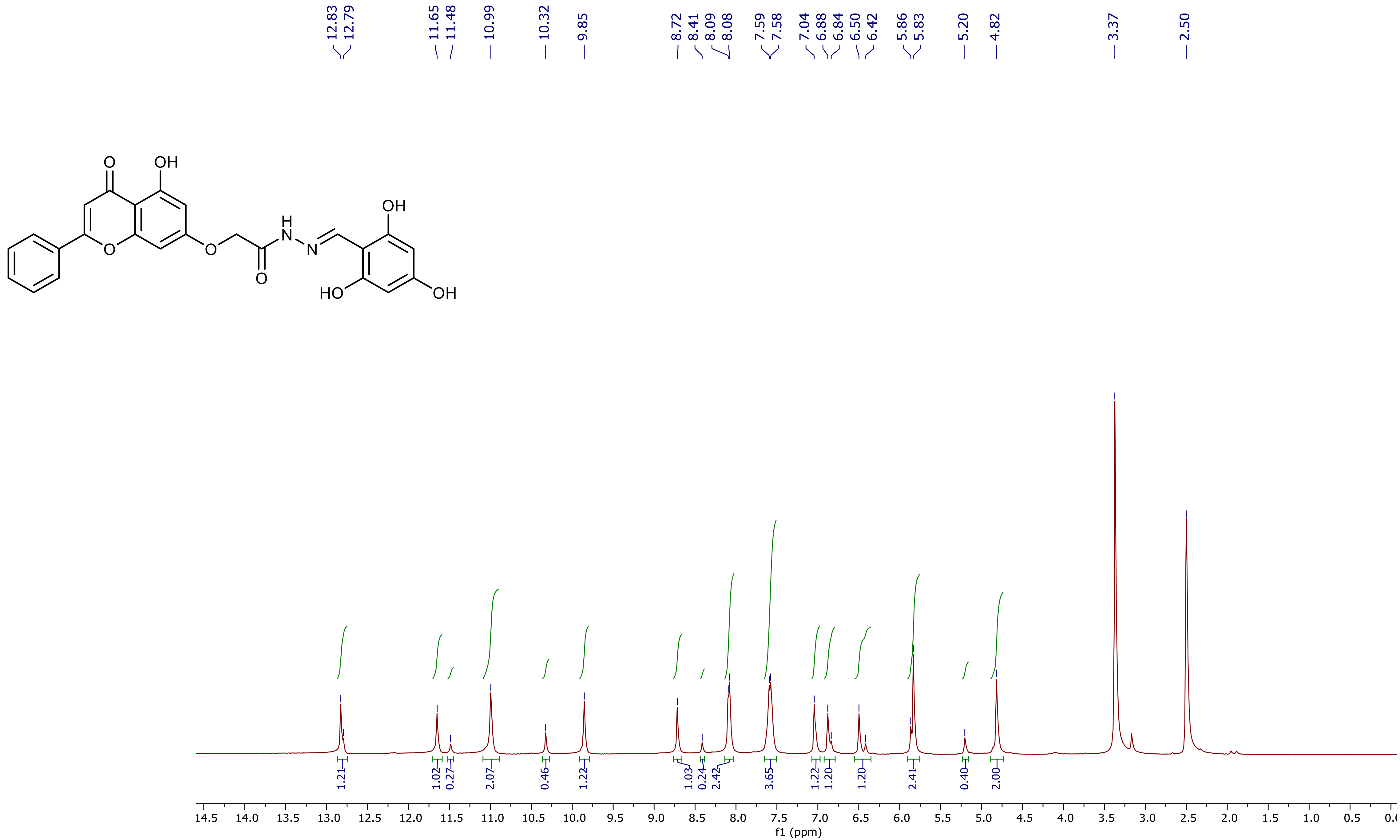

S21. ^{1}H NMR of Compound **4j**

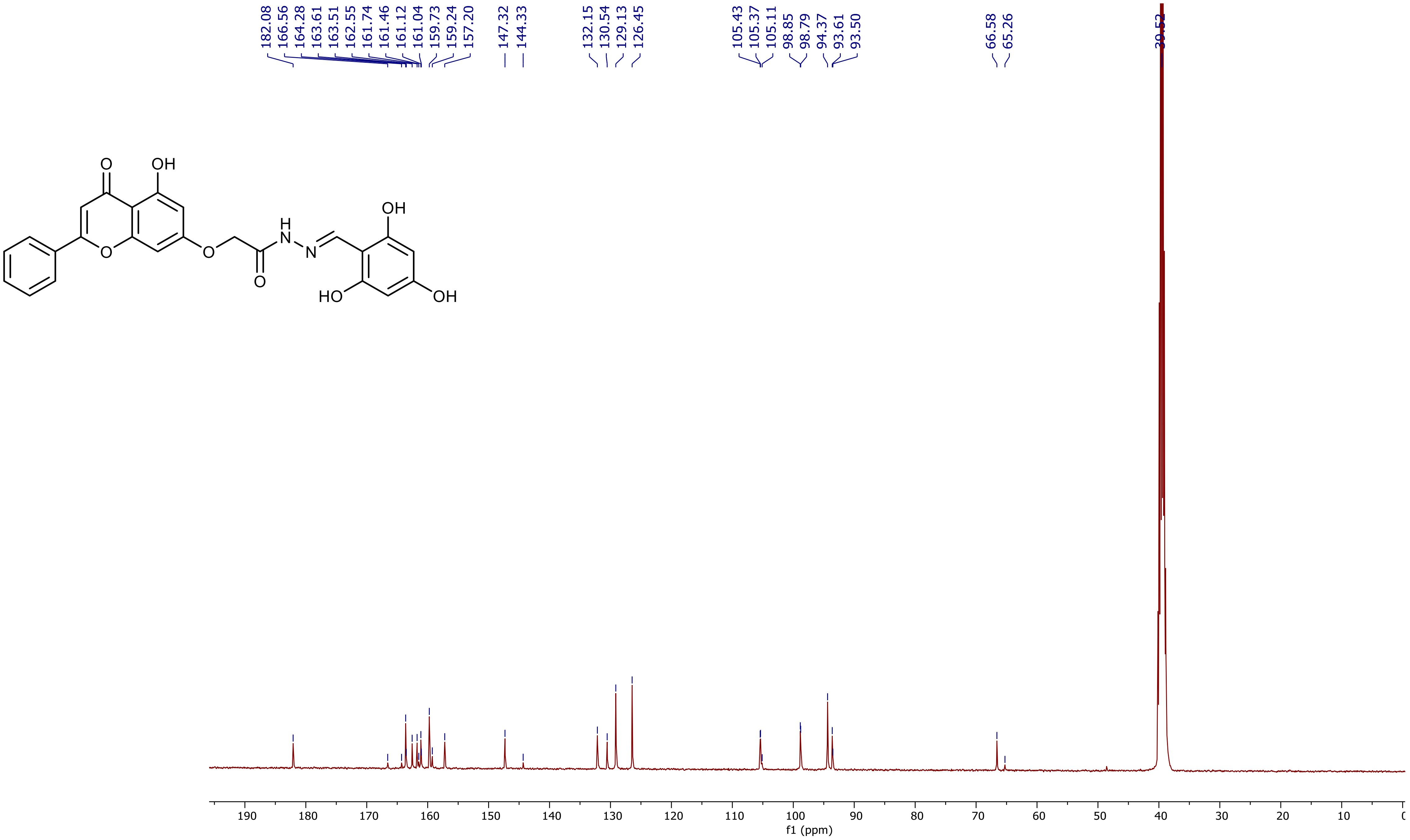

S22. ^{13}C NMR of Compound **4j**

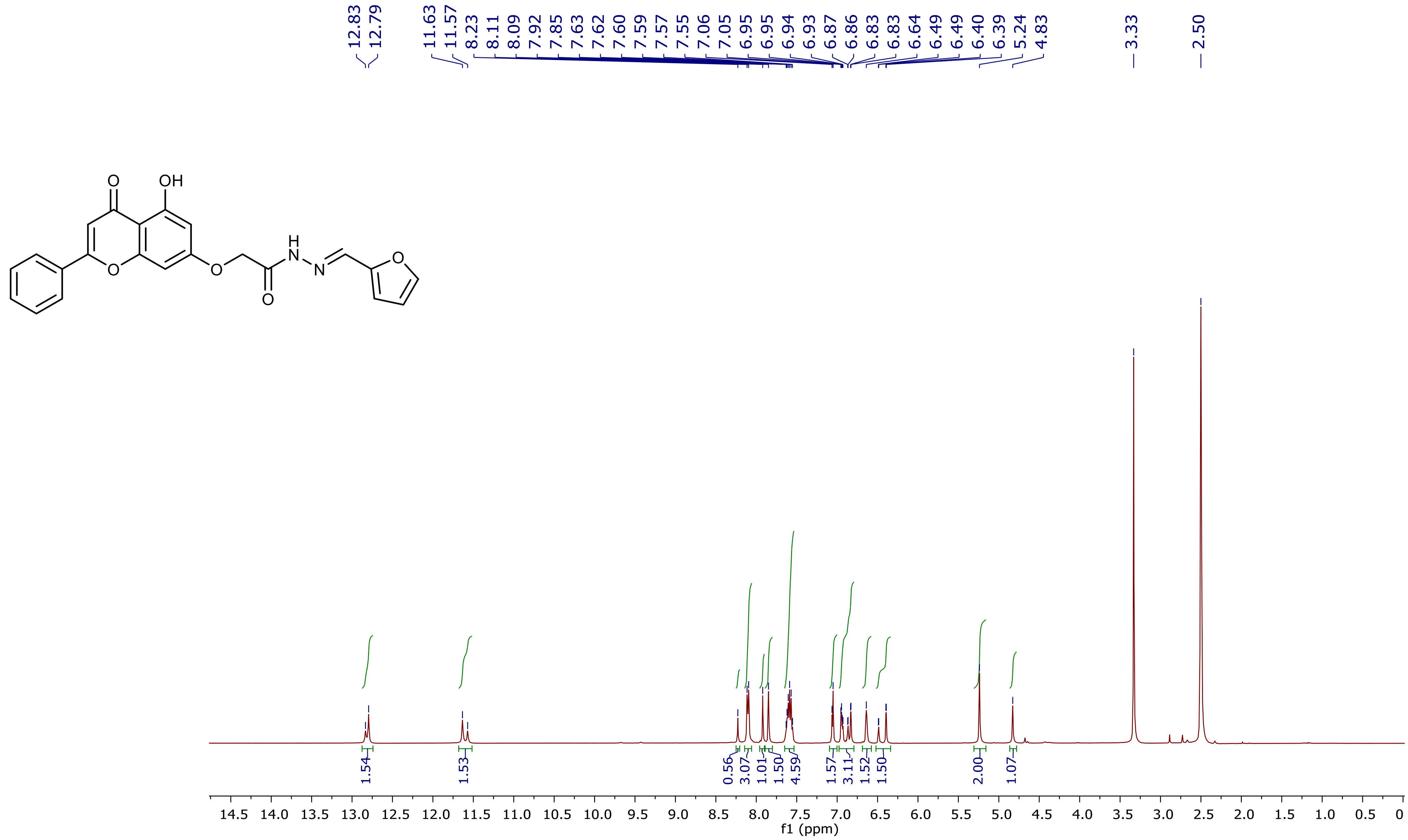

S23. ^{1}H NMR of Compound **4k**

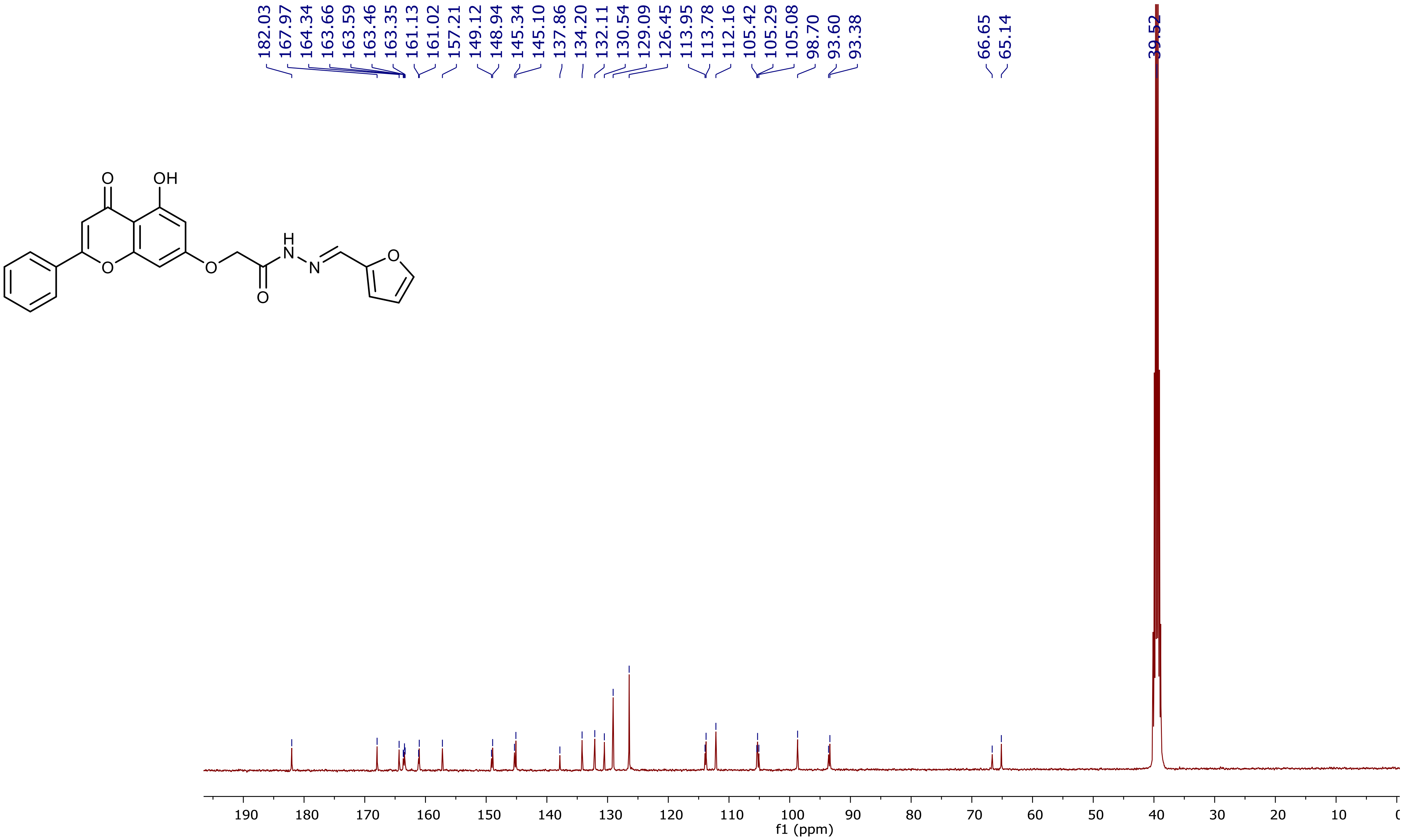

S24. ^{13}C NMR of Compound **4k**

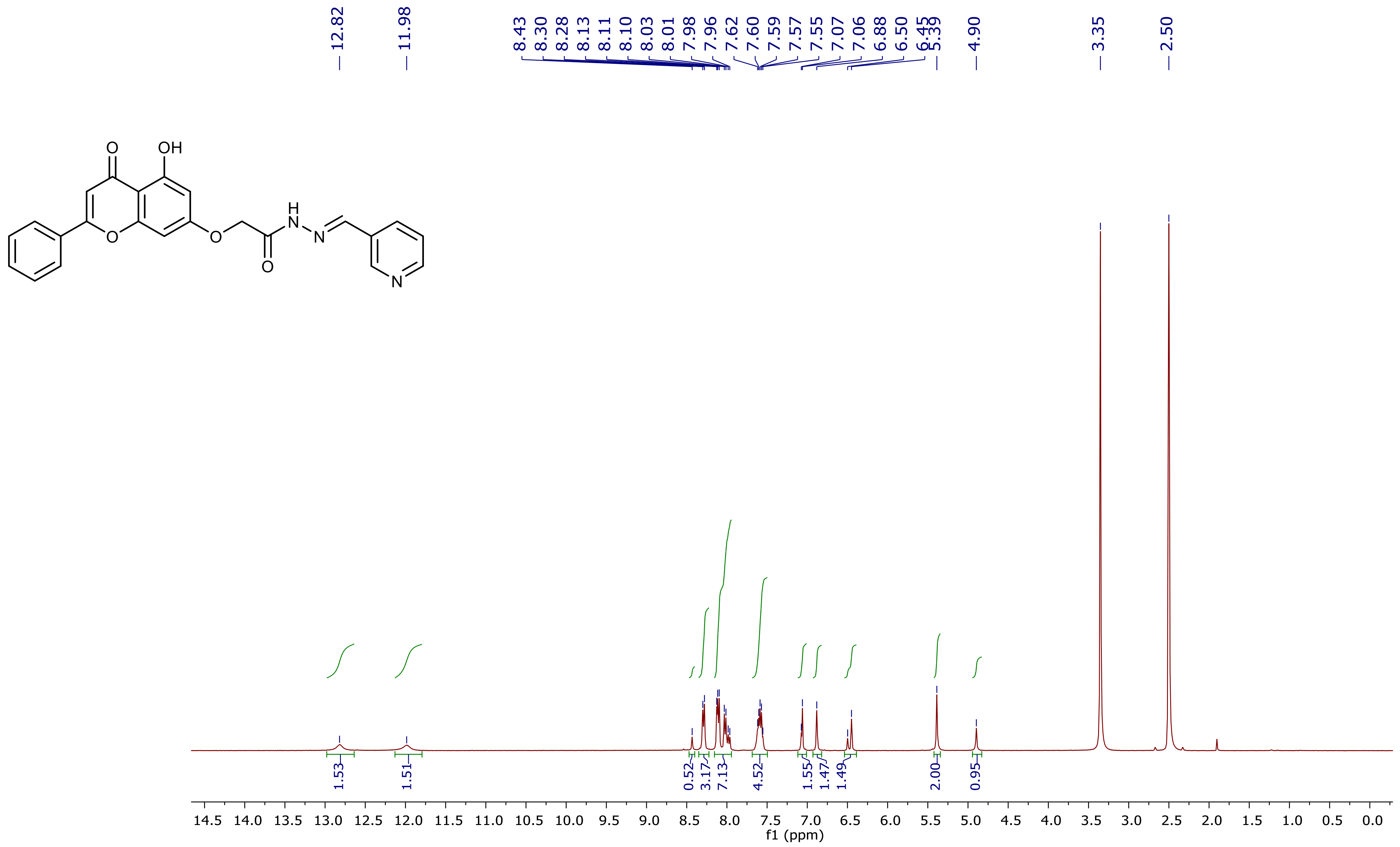

S25. ^{1}H NMR of Compound **4l**

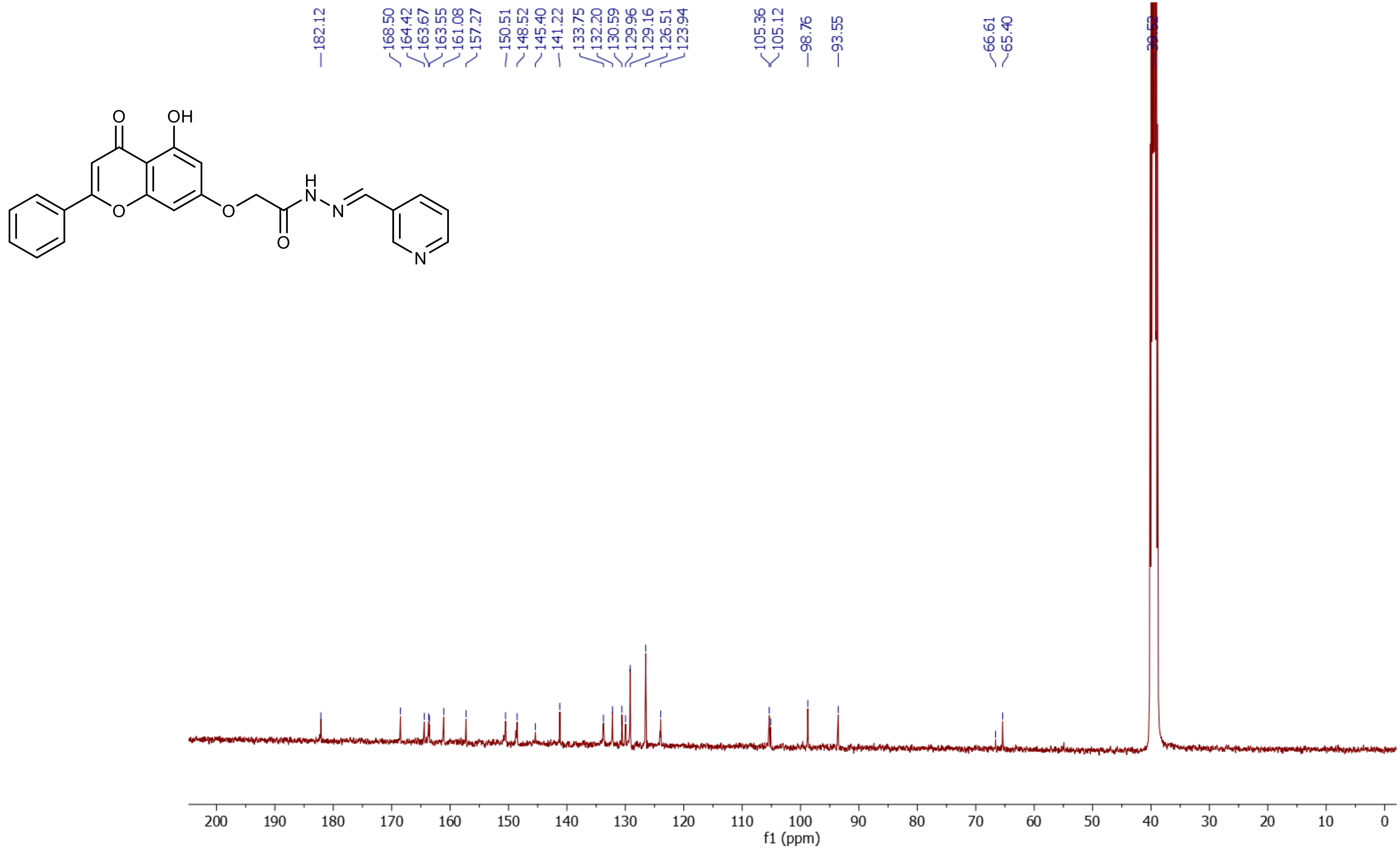

S26. ^{13}C NMR of Compound **4l**

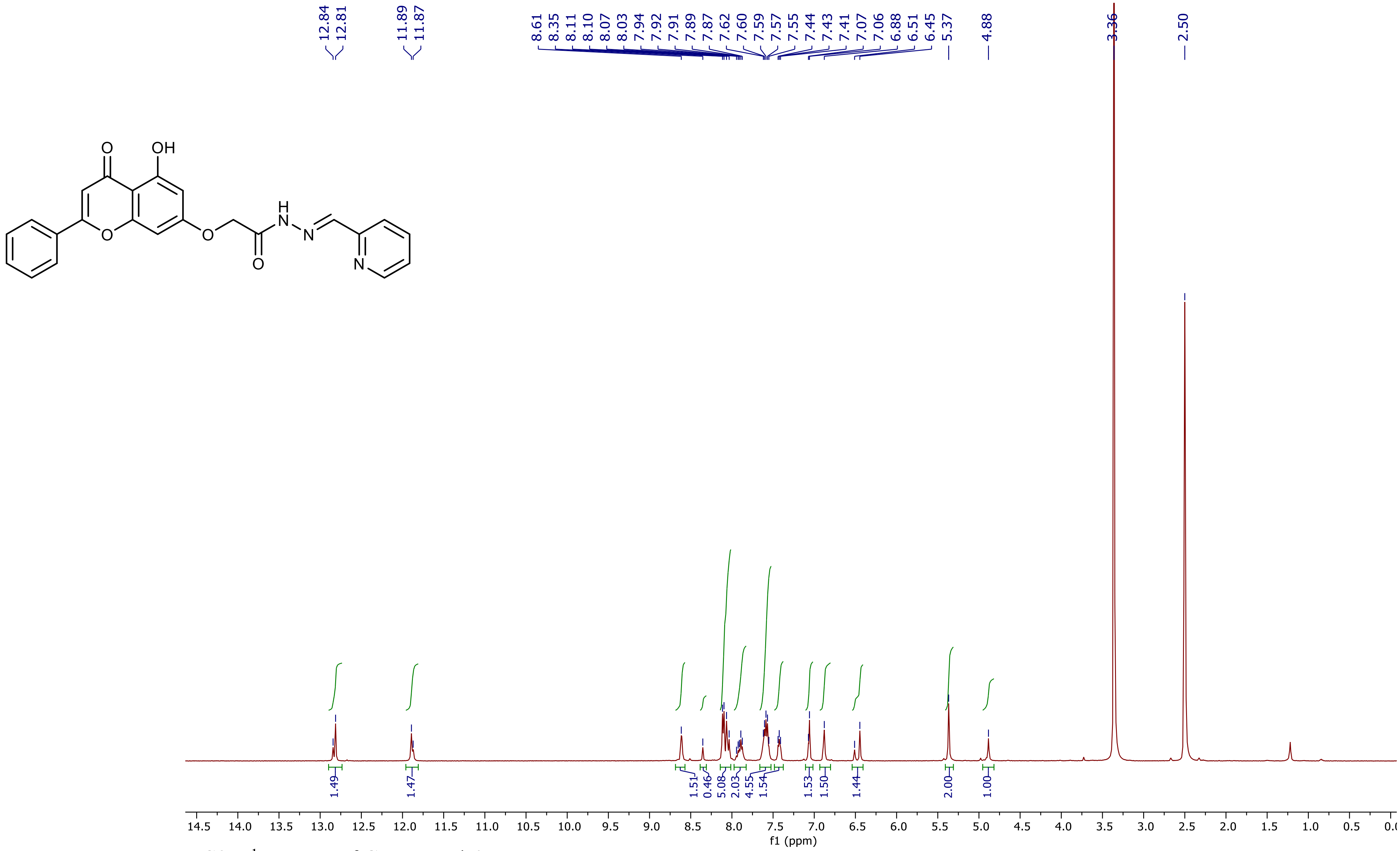

S27. ^{1}H NMR of Compound **4m**

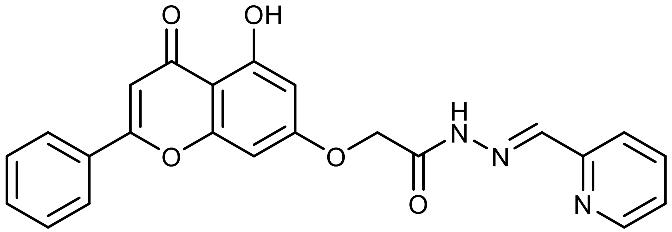

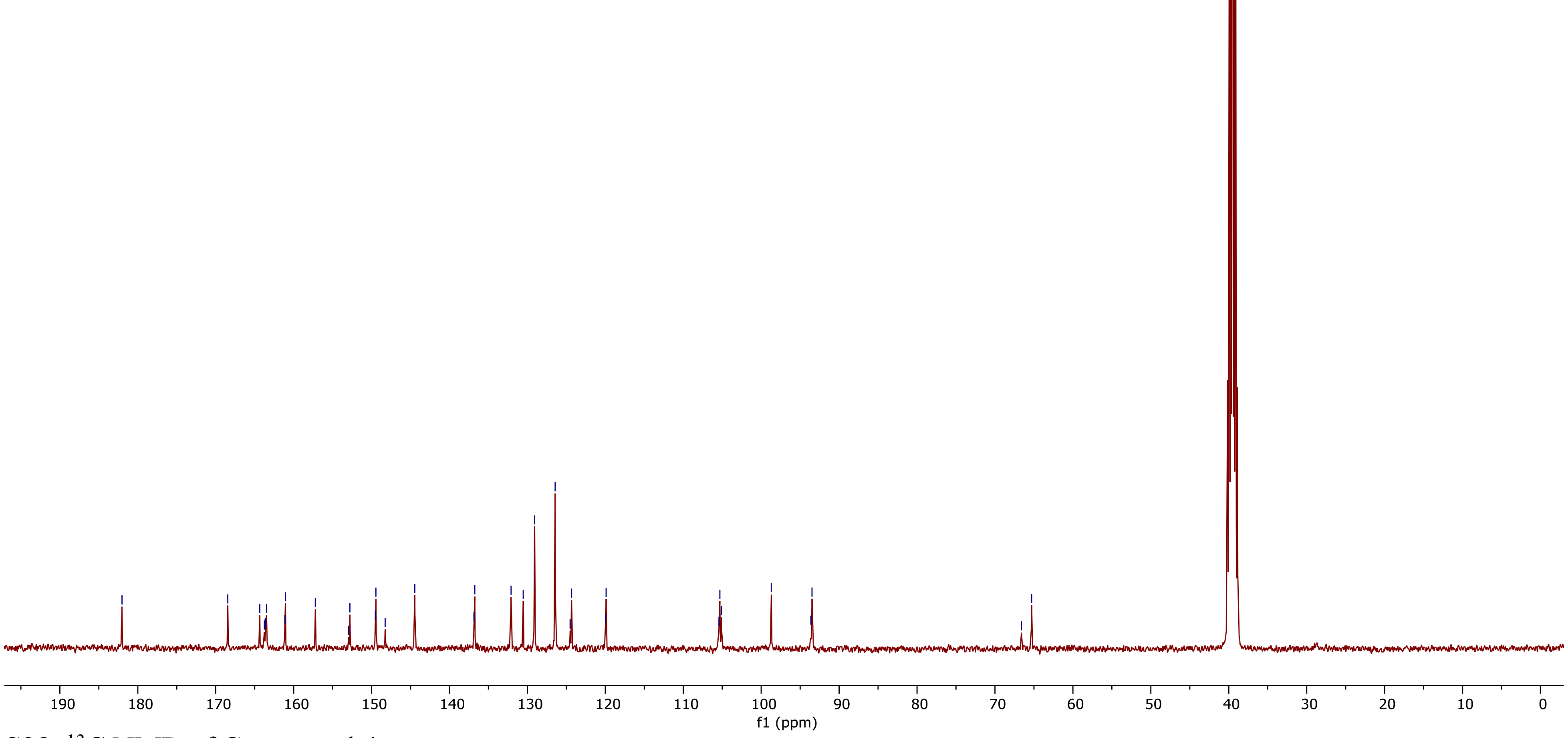

S28. ^{13}C NMR of Compound **4m**

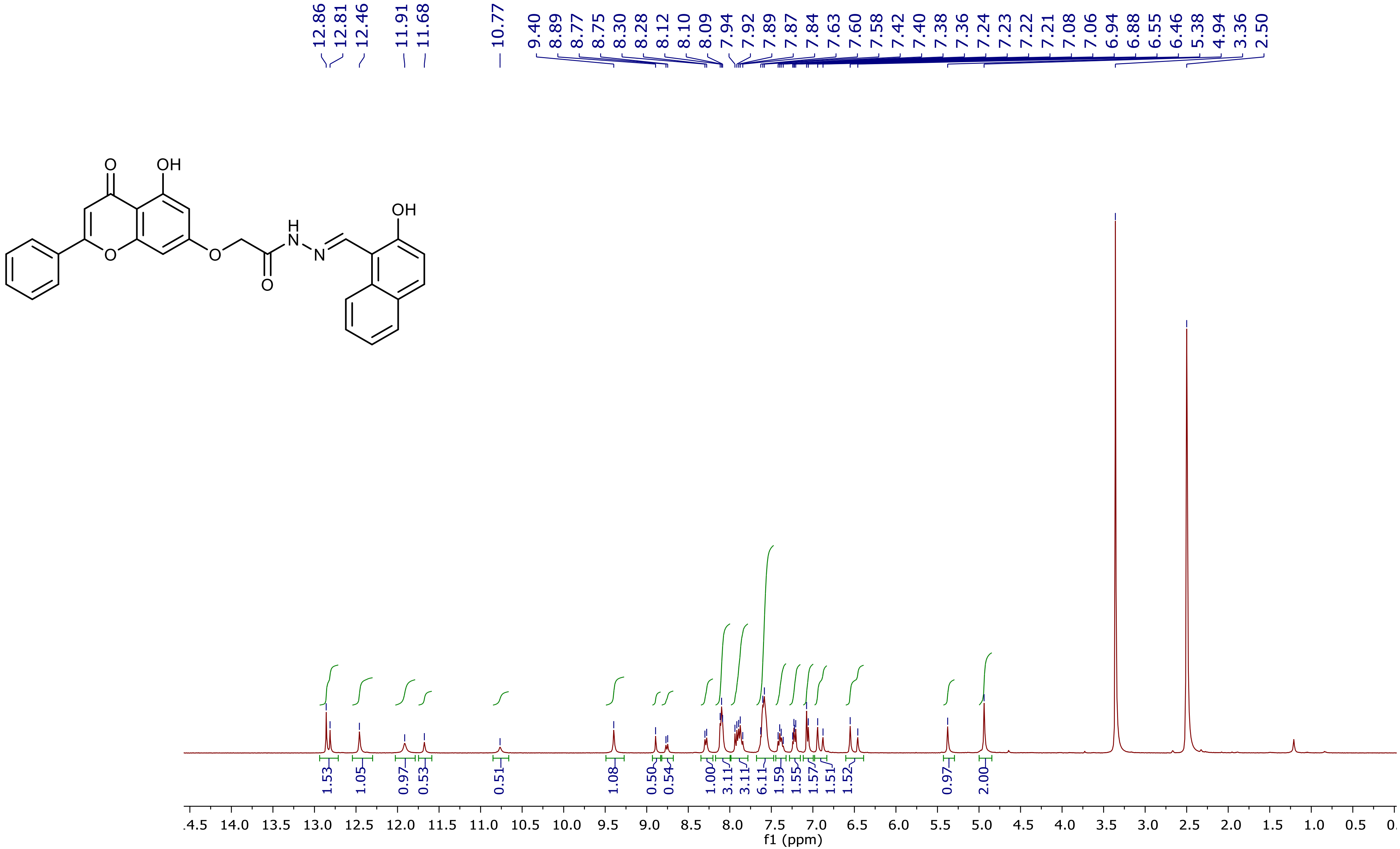

S29. ^{1}H NMR of Compound **4n**

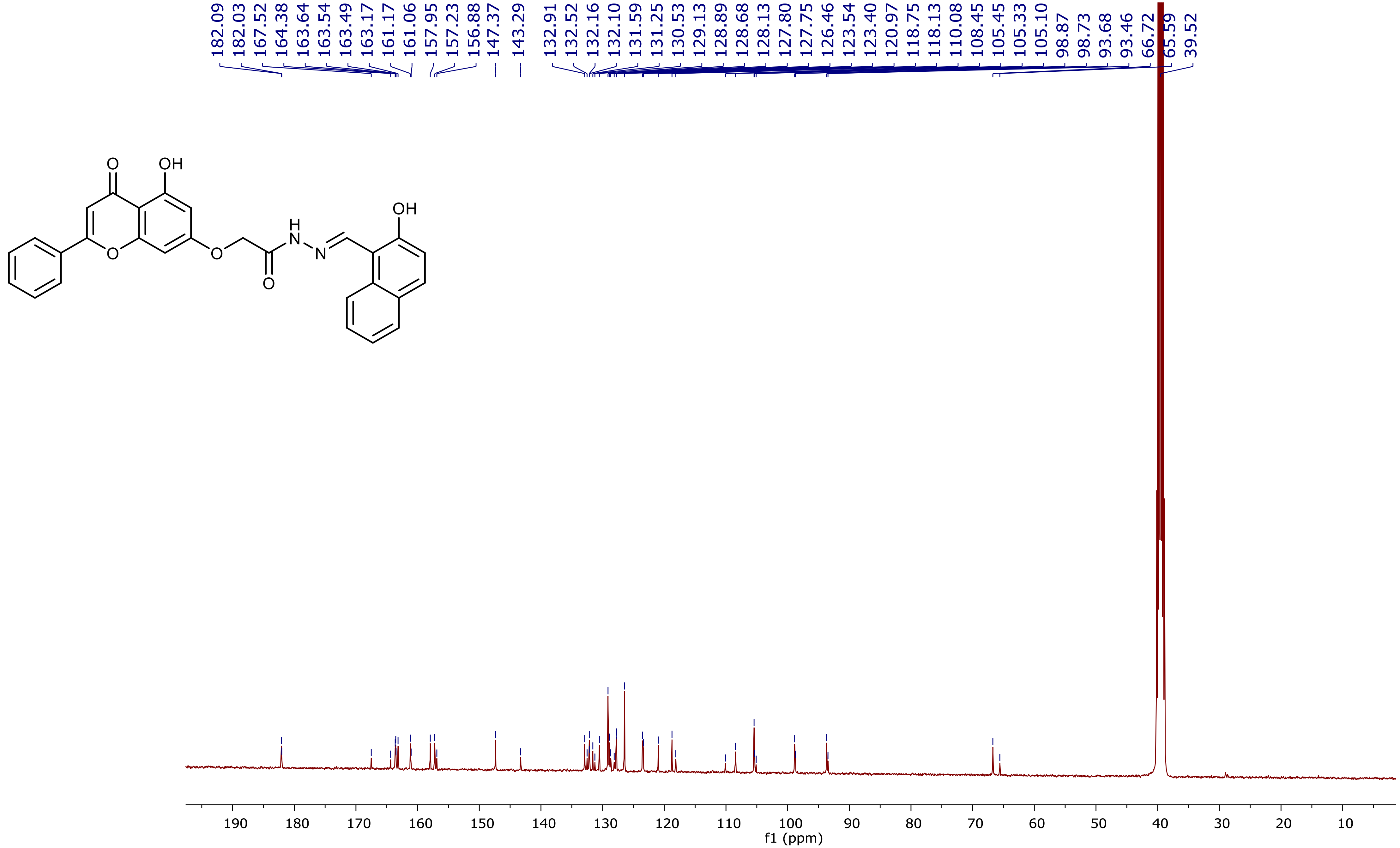

S30. ^{13}C NMR of Compound **4n**

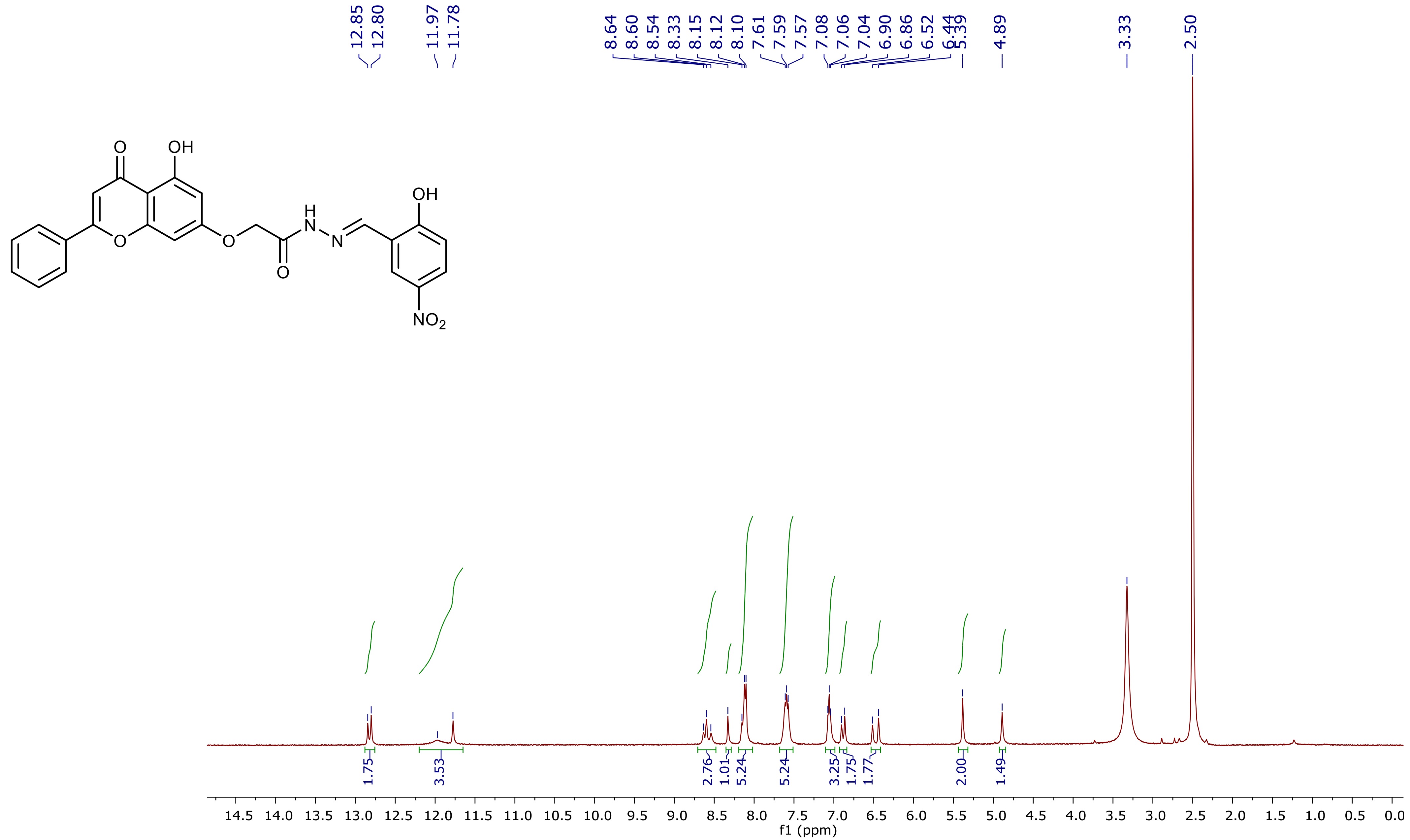

S31. ^{1}H NMR of Compound **4o**

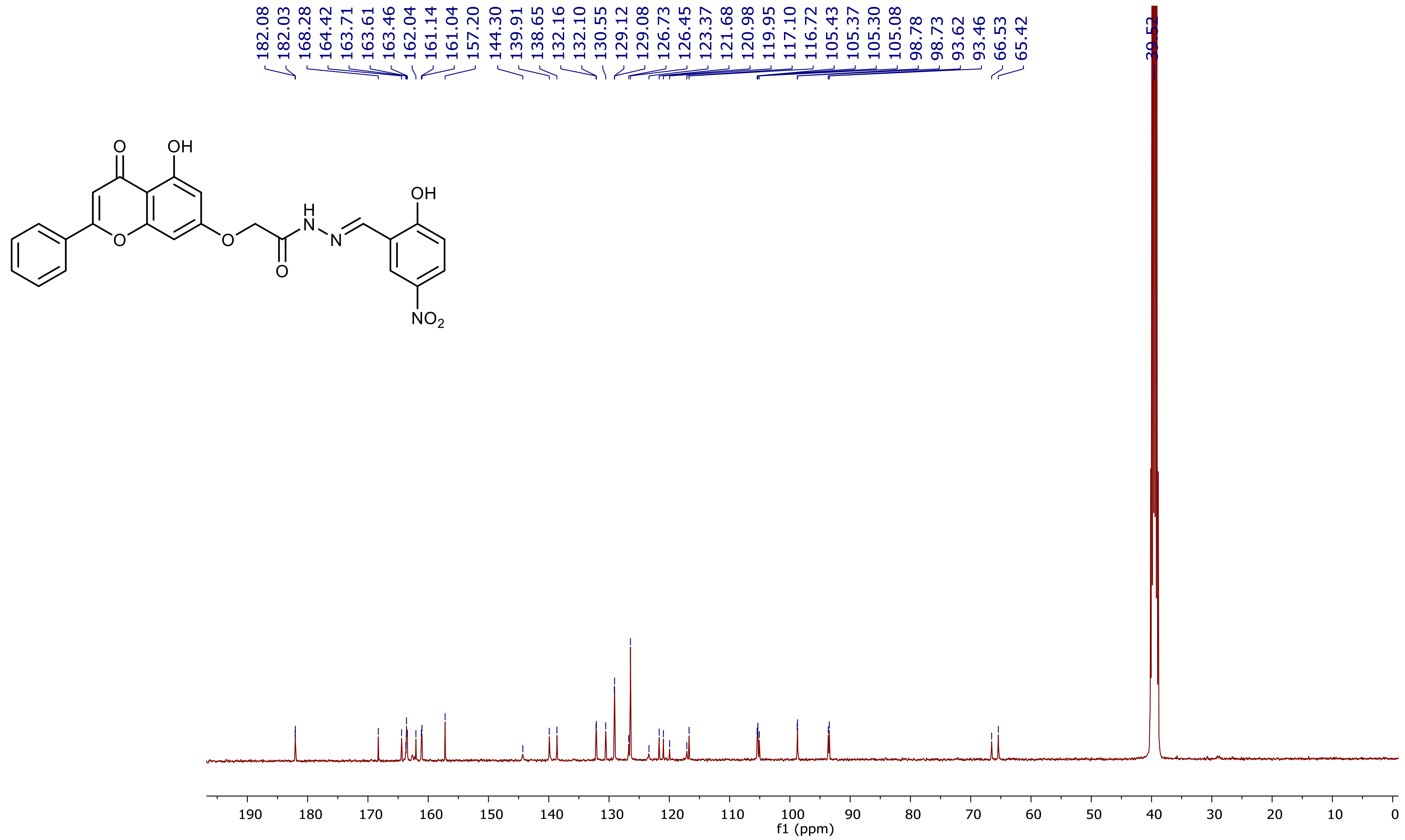

S32. ^{13}C NMR of Compound **4o**
